# Sacral Neural Crest‐Derived Extracellular Vesicles Promote Pelvic Ganglion Repair Through Anti‐Pyroptotic Effects Involving ANXA1–FPR2 Signaling

**DOI:** 10.1002/advs.78321

**Published:** 2026-10-15

**Authors:** Ting Yang, Wenwei Liu, Hufei Wang, Yuxin Wu, Yuanchen Ma, Xiaofeng Wang, Tianxiang Lei, Qiao Tang, Yebohao Zhou, Weiqiang Li, Andy Peng Xiang, Wu Song

**Affiliations:** ^1^ Department of Gastrointestinal Surgery The First Affiliated Hospital Sun Yat‐sen University Guangzhou Guangdong China; ^2^ Center For Stem Cell Biology and Tissue Engineering Key Laboratory for Stem Cells and Tissue Engineering Ministry of Education, Zhongshan School of Medicine Sun Yat‐sen University Guangzhou Guangdong China; ^3^ Department of Thyroid and Hernia Surgery Guangdong Provincial People's Hospital Southern Medical University Guangzhou Guangdong China

**Keywords:** colorectal dysfunction, extracellular vesicles, major pelvic ganglia, pyroptosis, sacral neural crest cells

## Abstract

Pelvic autonomic nerve injury is a major cause of postoperative bowel dysfunction, yet effective strategies for restoring damaged pelvic ganglia remain lacking. Here, we demonstrate that extracellular vesicles derived from human pluripotent stem cell‐derived sacral neural crest cells (Sacral NC‐EVs) promote pelvic neuroregeneration by suppressing oxidative stress‐induced pyroptosis. In a rat model of major pelvic ganglion (MPG) injury, local administration of Sacral NC‐EVs improved gastrointestinal motility and partially restored rectal neural innervation and epithelial barrier integrity. Mechanistic analyses showed that MPG injury increased oxidative stress and activated the NLRP3‐caspase‐1‐GSDMD pyroptosis pathway, whereas Sacral NC‐EVs attenuated both responses. Comparisons across EV sources showed that Sacral NC‐EVs more effectively suppressed oxidative stress and pyroptosis and increased PGP9.5‐positive and ChAT‐positive neuronal areas than mesenchymal stem cell‐derived EVs and vagal neural crest‐derived EVs. Proteomic and functional analyses implicated EV‐associated annexin A1 (ANXA1) as one contributor to the antioxidant and anti‐pyroptotic effects of Sacral NC‐EVs through FPR2 signaling. Collectively, our findings support Sacral NC‐EVs as a promising cell‐free strategy for pelvic neural repair by targeting the oxidative stress‐pyroptosis axis.

## Graphical abstract

Extracellular vesicles derived from human pluripotent stem cell‐derived sacral neural crest cells promote repair of injured major pelvic ganglia and improve colorectal function. These vesicles reduce oxidative stress and pyroptosis, with ANXA1–FPR2 signaling contributing to their protective effects, and show a favorable therapeutic profile compared with those derived from mesenchymal stem cells and vagal neural crest cells.

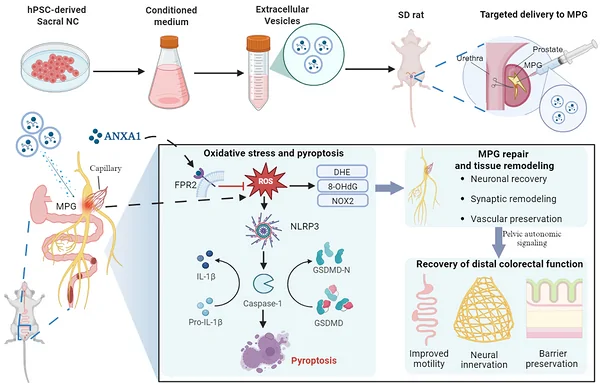

## 1 Introduction

Pelvic autonomic nerve injury is a common and often unavoidable complication of pelvic surgery, particularly during radical procedures for rectal, prostate, and gynecologic cancers. Mechanical stretching, compression, thermal injury, and vascular disruption during pelvic dissection can damage the pelvic autonomic plexus, leading to persistent postoperative bowel dysfunction [1, 2, 3, 4]. The major pelvic ganglion (MPG) is a key autonomic relay that provides innervation to pelvic organs, including the rectum, bladder, and reproductive organs [1]. Disruption of this structure not only compromises direct autonomic innervation of intestinal smooth muscle and vasculature but also impairs extrinsic regulation of the enteric nervous system (ENS), thereby disturbing colorectal motility, secretion, and epithelial homeostasis [5, 6, 7]. Current clinical management remains largely symptomatic and does not directly restore damaged pelvic autonomic nerves or reconstruct the disrupted gut‐neural axis [8]. Thus, regenerative strategies that specifically target MPG injury are urgently needed.

Neural repair after pelvic autonomic injury is further limited by the hostile local microenvironment created by surgical trauma. Damage to the perineural vasculature can induce ischemia, hypoxia, and excessive oxidative stress, all of which are known to exacerbate neuronal degeneration and impair axonal regeneration [9]. Increasing evidence from both central and peripheral nervous system injury models suggests that oxidative stress‐driven inflammatory cascades, particularly inflammasome activation and pyroptosis, are key contributors to neuronal degeneration [10, 11, 12, 13]. These pathological processes not only exacerbate neuronal loss but also inhibit axonal regeneration and synaptic remodeling, thereby severely limiting functional recovery [14]. However, whether oxidative stress‐induced pyroptosis occurs in the MPG and limits its regeneration after injury remains unclear.

Sacral neural crest cells (Sacral NC) contribute to the embryonic formation of pelvic autonomic ganglia [15, 16]. This developmental relationship provides a biological rationale for evaluating Sacral NC‐derived products in pelvic autonomic nerve repair. Although stem cell‐based therapies have shown promise for neural regeneration, their clinical translation remains limited by biological and safety‐related challenges, including poor cell engraftment and survival, immune rejection, uncontrolled differentiation, and tumorigenic potential, as well as practical barriers associated with scalable manufacturing, stringent quality control, long‐term storage, and transportation of viable cell products [17, 18, 19]. Extracellular vesicles (EVs), as key mediators of stem cell paracrine signaling, have therefore emerged as a promising cell‐free therapeutic modality with potential advantages in product stability, handling, quality control, and reproducible delivery of bioactive cargo [20, 21]. Importantly, EVs constitute a membrane‐enclosed and experimentally isolatable bioactive fraction of the broader secretome [20]. In contrast to soluble secreted factors in conditioned medium, which are prone to enzymatic degradation, rapid diffusion, and loss of bioactivity within the tissue microenvironment, EVs are protected by a lipid bilayer that preserves bioactive proteins, lipids, and nucleic acids [22, 23]. This structural organization enables stable cargo presentation and intercellular communication through surface receptor engagement, endocytic uptake, and other membrane‐dependent interactions, thereby providing conceptual advantages over the use of bulk secretome for regenerative therapy [23, 24].

The therapeutic properties of EVs may be particularly advantageous in the context of local administration for peripheral neural repair. Local delivery allows EVs to act directly within the injured MPG microenvironment, increasing local bioavailability while reducing systemic dispersion and off‐target exposure [25]. Moreover, the nanoscale size and membrane properties of EVs may favor retention within extracellular matrix‐rich tissues and facilitate uptake by resident neural and glial cells [26]. These features are especially relevant to the MPG, an anatomically confined autonomic ganglion characterized by dense neuron‐glia interactions and limited intrinsic regenerative capacity [27]. Because EV cargo is shaped by donor‐cell identity, the developmental relationship between Sacral NC and pelvic autonomic ganglia supports evaluating Sacral NC as an EV source for pelvic nerve repair [25, 28]. Whether Sacral NC‐EVs offer greater therapeutic benefit than EVs from other sources remains unknown.

In this study, we investigated whether EVs derived from human pluripotent stem cell (hPSC)‐induced Sacral NC could promote repair of the injured MPG as a cell‐free therapeutic strategy. We further examined whether restoration of MPG integrity could drive downstream recovery of intestinal structure and function. We next focused on oxidative stress‐driven pyroptosis as a key injury mechanism that limits MPG repair and a pathological axis potentially attenuated by Sacral NC‐EVs. To compare therapeutic activity across donor‐cell sources, we evaluated Sacral NC‐EVs alongside mesenchymal stem cell‐derived EVs (MSC‐EVs) and vagal neural crest cell‐derived EVs (Vagal NC‐EVs). MSC‐EVs served as a widely studied regenerative comparator, whereas Vagal NC‐EVs provided a neural crest‐derived comparator with a distinct axial identity. Finally, integrated EV proteomics and functional perturbation studies were used to evaluate ANXA1 as a bioactive Sacral NC‐EV cargo and to define its contribution to MPG repair through FPR2‐dependent suppression of oxidative stress and pyroptosis.

## 2 Results

### 2.1 Sacral NC‐EVs Improve Intestinal Dysfunction Following MPG Injury

To determine whether pelvic surgery is associated with rectal autonomic denervation, rectal tissue specimens were collected from patients with or without a history of pelvic surgery involving the pelvic autonomic nerve region. Immunohistochemical analysis showed a marked reduction in the pan‐neuronal marker βIII‐tubulin (TUJ1) in the Injury group, accompanied by significant decreases in ChAT, nNOS, TH, and VIP expression (Figures 1A–D and S1A–C). Consistently, immunofluorescence staining revealed broad loss of neuronal fibers across multiple autonomic neuronal subtypes (Figures 1E–G and S1D–F), indicating that pelvic surgery is associated with extensive rectal autonomic denervation.

**FIGURE 1:**
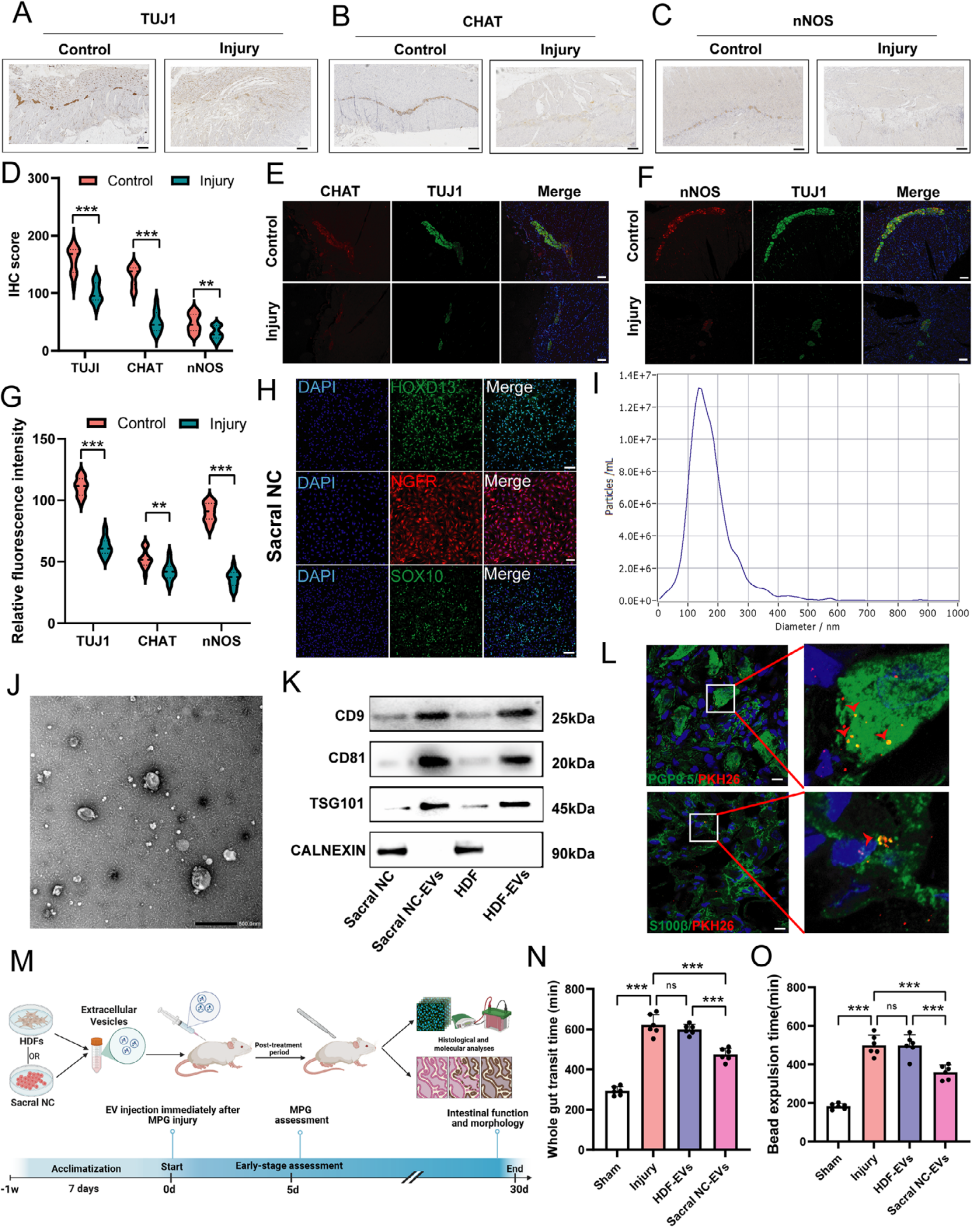
Sacral NC‐EVs improve intestinal dysfunction following MPG injury. (A–C) Representative immunohistochemical staining of TUJ1 (A), ChAT (B), and nNOS (C) in rectal muscularis propria from Control patients without prior pelvic surgery and Injury patients with a history of pelvic surgery involving pelvic autonomic nerve regions. Scale bars, 100 µm. (D) Semi‐quantitative H‐score analysis of TUJ1, ChAT, and nNOS immunohistochemical staining in the Control and Injury groups; *n* = 15 independent human specimens per group. (E,F) Representative immunofluorescence staining of ChAT (E) and nNOS (F) in rectal tissues from Control and Injury groups. Scale bars, 100 µm. (G) Quantification of relative fluorescence intensity for TUJ1, ChAT, and nNOS; *n* = 15 independent human specimens per group. (H) Immunofluorescence staining of HOXD13, NGFR, and SOX10 in hPSC‐derived Sacral NC. Scale bars, 50 µm. (I) Nanoparticle tracking analysis showing the size distribution of a diluted Sacral NC‐EV sample. (J) Transmission electron microscopy image showing the morphology of Sacral NC‐EVs. Scale bar, 500 nm. (K) Western blot analysis of EV‐positive markers CD9, CD81, and TSG101 and the negative marker CALNEXIN in Sacral NC, Sacral NC‐EVs, HDFs, and HDF‐EVs. (L) Representative confocal images showing the localization of PKH26‐labeled Sacral NC‐EVs in the injured MPG. PKH26 signals were spatially associated with PGP9.5^+^ neuronal structures and S100β^+^ glial cells. Scale bars, 10 µm. (M) Schematic illustration of the experimental design for EV administration in the rat MPG injury model. (N,O) Whole‐gut transit time (N) and bead expulsion time (O) in Sham, Injury, HDF‐EVs, and Sacral NC‐EVs groups; *n* = 5 rats per group. Violin plots show data distribution with median and quartiles. Bar graphs are presented as mean ± SD. Each dot represents one independent human specimen or animal. *p* values were calculated using a two‐sided Mann–Whitney *U* test for two‐group comparisons of human tissue staining measurements (D,G) and one‐way ANOVA followed by Tukey's multiple comparisons test for comparisons among multiple animal groups (N,O). ns, not significant; ^*^ *p* < 0.05, ^**^ *p* < 0.01, ^***^ *p* < 0.001.

Given the developmental relationship between Sacral NC and pelvic autonomic ganglia, we next generated Sacral NC from hPSCs and confirmed their identity. Sacral NC expressed the neural crest markers SOX10 and NGFR, together with the sacral‐associated marker HOXD13 (Figure 1H) [29]. qPCR analysis further confirmed downregulation of the pluripotency marker *NANOG* and upregulation of the neural crest markers *SOX10* and *NGFR* in Sacral NC compared with hPSCs (Figure S1G). Additional qPCR profiling revealed a posterior HOX expression pattern, with increased expression of *HOXB8*, *HOXD8*, and *HOXC9* and robust induction of the more caudal, sacral‐associated genes *HOXB13* and *HOXD13*, consistent with the acquisition of sacral axial identity (Figure S1H).

EVs were isolated from Sacral NC‐conditioned medium and characterized. In parallel, human dermal fibroblast‐derived EVs (HDF‐EVs) were included as a non‐neural somatic EV comparator representing a donor‐cell source distinct from Sacral NC [30]. Nanoparticle tracking analysis and transmission electron microscopy showed a size distribution and vesicular morphology consistent with small EVs (Figures 1I,J and S1I). Western blotting confirmed enrichment of EV‐associated markers CD9, CD81, and TSG101, whereas CALNEXIN was absent or markedly depleted in EV preparations compared with parental cells (Figure 1K). After local administration, PKH26‐labeled Sacral NC‐EVs were detected within the injured MPG and were spatially associated with PGP9.5^+^ neuronal cells and S100β^+^ glial cells, with little colocalization with CD68^+^ macrophages (Figures 1L and S1J).

To evaluate therapeutic efficacy in vivo, bilateral MPG injury was induced, followed immediately by local multipoint peri‐ganglionic EV administration (Figure 1M). Each MPG side received 10 µL of EV suspension standardized to a common nominal total‐protein input of approximately 10 µg. NTA characterization of the administration formulations yielded estimated particle inputs of approximately 9.7 × 10^10^ HDF‐EV particles and 8.6 × 10^10^ Sacral NC‐EV particles per MPG side, respectively. Thus, EV administration was standardized by nominal protein input, and the NTA‐estimated particle inputs were broadly comparable between the HDF‐EV and Sacral NC‐EV formulations. MPG injury induced severe intestinal dysfunction, as evidenced by prolonged whole‐gut transit time, delayed bead expulsion, reduced fecal pellet output, and decreased fecal water content (Figures 1N,O and S1K,L). Sacral NC‐EV treatment significantly improved these functional parameters, whereas HDF‐EVs showed limited or no therapeutic benefit under the same conditions (Figures 1N,O and S1K,L). Under identical labeling, administration, and imaging conditions, PKH26‐labeled HDF‐EVs and Sacral NC‐EVs exhibited comparable tissue‐level distribution within PGP9.5^+^ regions of the injured MPG (Figure S1M,N). Therefore, the greater functional benefit observed with Sacral NC‐EVs was unlikely to be explained primarily by differences in their gross local distribution. Collectively, these findings demonstrate that hPSC‐derived Sacral NC produce well‐characterized EVs that localize to the injured MPG and ameliorate MPG injury‐induced intestinal dysfunction.

### 2.2 Sacral NC‐EVs Attenuate MPG Injury–Induced Rectal Denervation

Pelvic autonomic innervation is essential for distal pelvic organ function, including the regulation of rectal motility and intestinal homeostasis [1, 4]. To determine whether MPG injury contributes to rectal neural disruption, we examined the myenteric plexus within the rectal muscular layer of MPG‐injured rats. MPG injury caused a pronounced loss of TUJ1^+^ nerve fibers and a significant reduction in NeuN^+^ neuronal area within the rectal myenteric plexus (Figure 2A–C). Sacral NC‐EV treatment partially restored both TUJ1^+^ and NeuN^+^ areas compared with injured controls, whereas HDF‐EVs showed only limited improvement under the same conditions (Figure 2A–C).

**FIGURE 2:**
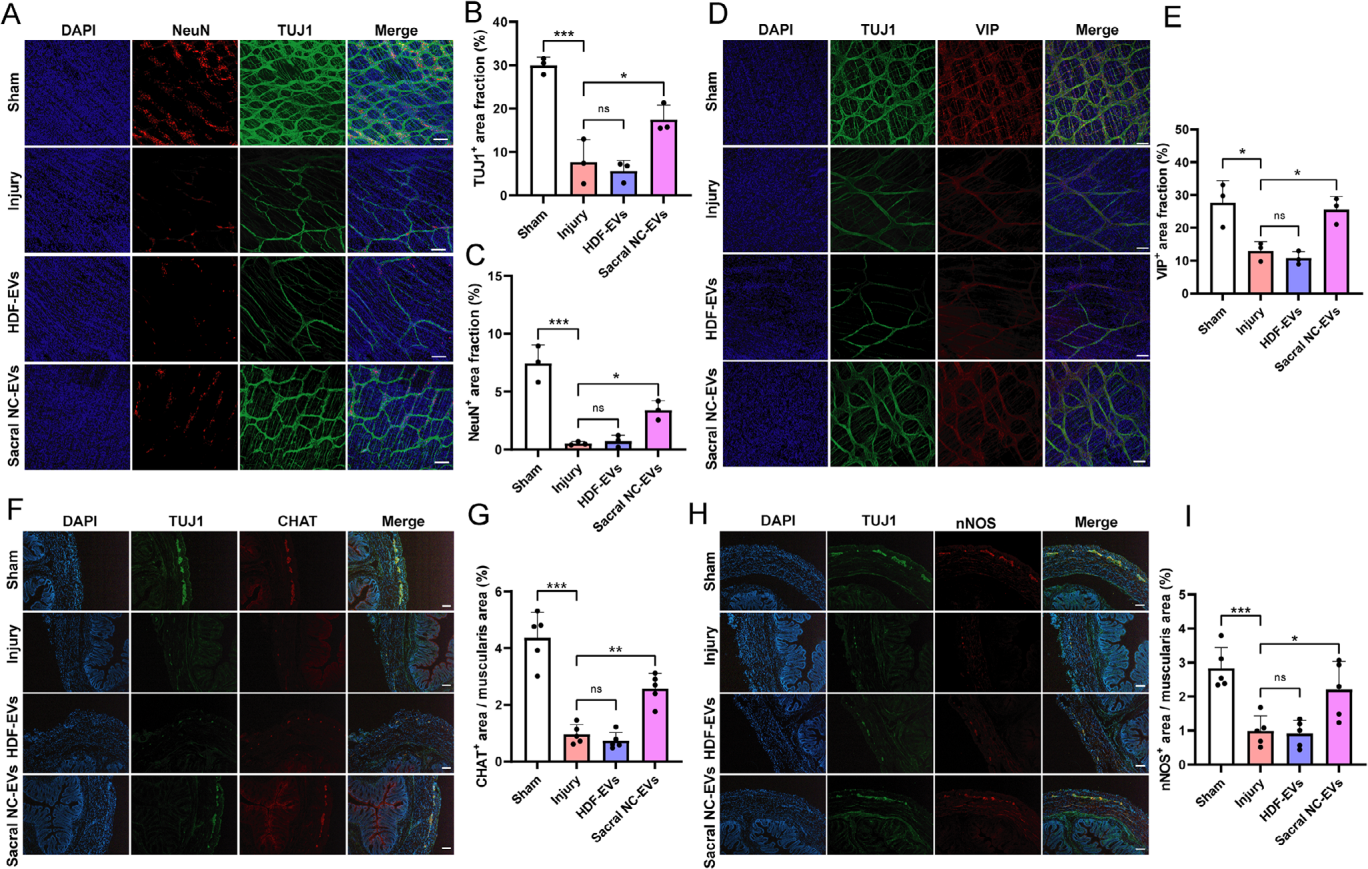
Sacral NC‐EVs attenuate MPG injury‐induced rectal denervation. (A) Representative immunofluorescence staining of NeuN and TUJ1 in rectal myenteric plexus preparations from Sham, Injury, HDF‐EVs, and Sacral NC‐EVs groups. Scale bars, 100 µm. (B,C) Quantification of TUJ1^+^ area fraction (B) and NeuN^+^ area fraction (C) in the rectal myenteric plexus; *n* = 3 rats per group. (D) Representative immunofluorescence staining of VIP in rectal myenteric plexus preparations from each group. Scale bars, 100 µm. (E) Quantification of VIP^+^ area fraction in the rectal myenteric plexus; *n* = 3 rats per group. (F) Representative immunofluorescence staining of ChAT in rectal transverse sections from each group. Scale bars, 100 µm. (G) Quantification of ChAT^+^ area as a percentage of the muscularis area; *n* = 5 rats per group. (H) Representative immunofluorescence staining of nNOS in rectal transverse sections from each group. Scale bars, 100 µm. (I) Quantification of nNOS^+^ area as a percentage of the muscularis area; *n* = 5 rats per group. Data are presented as mean ± SD. Each dot represents one independent animal. *p* values were calculated using one‐way ANOVA followed by Tukey's multiple‐comparison test. ns, not significant; ^*^ *p *< 0.05, ^**^ *p* < 0.01, ^***^ *p* < 0.001.

Subtype‐specific analysis further revealed broad reductions in VIP^+^, nNOS^+^, TH^+^, and ChAT^+^ neuronal areas after MPG injury in rectal myenteric plexus preparations and transverse rectal sections (Figure 2D–I; Figures S2A–F and S3A–D). Sacral NC‐EVs significantly increased VIP^+^, TH^+^, and ChAT^+^ area fractions in myenteric plexus preparations, whereas the nNOS^+^ area showed an upward trend that did not reach statistical significance (Figures 2D,E and S2A–F). In transverse rectal sections, Sacral NC‐EVs significantly increased ChAT^+^, nNOS^+^, and VIP^+^ areas as percentages of the muscularis area, whereas TH^+^ area showed only a modest, non‐significant increase after treatment (Figures 2F–I and S3A–D).

Together, these findings demonstrate that MPG injury causes extensive rectal denervation involving both pan‐neuronal structures and multiple autonomic neuronal subtypes. Sacral NC‐EV treatment alleviates these denervation‐associated changes and partially preserves rectal neural innervation, whereas HDF‐EVs provide limited benefit under the same conditions.

### 2.3 Sacral NC‐EVs Attenuate Rectal Mucosal Barrier Disruption and Apoptosis‐Associated Signaling Following MPG Injury

The mucosal barrier of the distal intestine is critically dependent on intact pelvic autonomic innervation; therefore, evaluating colorectal barrier integrity is essential to determine the downstream consequences of MPG injury. Loss of the enteric neural plexus following MPG injury may disrupt neuro‐epithelial regulation in the colorectum and contribute to mucosal barrier impairment [31]. In addition, impaired distal gut motility results in fecal stasis and elevated intraluminal pressure, which further exacerbate barrier dysfunction. Our experimental results demonstrated that plasma FITC‐dextran levels were significantly increased in the MPG injury group, suggesting enhanced intestinal permeability (Figure 3A). Local administration of Sacral NC‐EVs significantly reduced FITC‐dextran leakage, whereas HDF‐EVs failed to confer appreciable protection (Figure 3A), supporting a greater protective effect of Sacral NC‐EVs than HDF‐EVs under the tested conditions.

**FIGURE 3:**
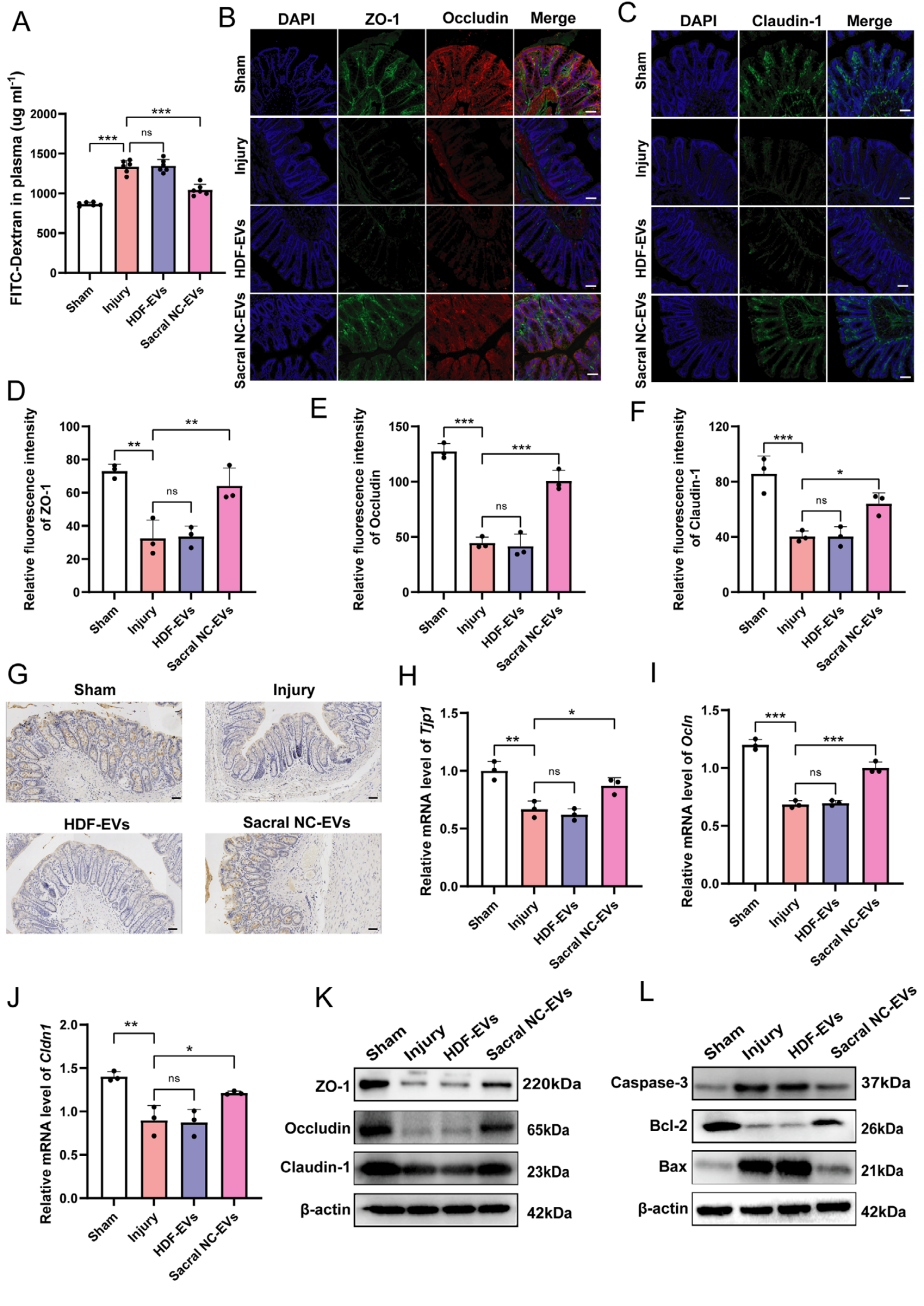
Sacral NC‐EVs attenuate rectal mucosal barrier disruption and epithelial apoptotic signaling following MPG injury. (A) Intestinal permeability assessed by plasma FITC‐dextran levels in Sham, Injury, HDF‐EVs, and Sacral NC‐EVs groups; *n* = 5 rats per group. (B,C) Representative immunofluorescence staining of ZO‐1/Occludin (B) and Claudin‐1 (C) in rectal mucosa from each group. Scale bars, 50 µm. (D–F) Quantification of relative fluorescence intensity for ZO‐1 (D), Occludin (E), and Claudin‐1 (F); *n* = 3 rats per group. (G) Representative immunohistochemical staining of MUC2 in rectal tissues from each group. Scale bars, 50 µm. (H–J) qPCR analysis of mRNA expression levels of *Tjp1* (H), *Ocln* (I), and *Cldn1* (J) in rectal tissues; *n* = 3 rats per group. (K) Western blot analysis of ZO‐1, Occludin, and Claudin‐1 protein levels in mucosa‐enriched rectal samples. (L) Western blot analysis of apoptosis‐related proteins, including caspase‐3, Bcl‐2, and Bax, in mucosa‐enriched rectal samples. Data are presented as mean ± SD for quantitative panels. Each dot represents one independent animal. *P* values were calculated using one‐way ANOVA followed by Tukey's multiple‐comparison test. ns, not significant; ^*^ *p *< 0.05, ^**^ *p *< 0.01, ^***^ *p *< 0.001.

To define the structural basis for barrier restoration, we examined epithelial tight junctions and the mucus layer in the rectum. In Sham‐operated animals, ZO‐1, Occludin, and Claudin‐1 displayed continuous, belt‐like localization at apical junctions, forming well‐organized tight junctions. MPG injury disrupted this architecture, producing fragmented and discontinuous staining with significantly decreased fluorescence intensity (Figure 3B–F). Similarly, MUC2 immunostaining revealed a dense and uniform mucus layer in Sham animals, whereas MPG injury caused thinning, patchy distribution, and decreased MUC2 signal (Figures 3G and S4A,B). Sacral NC‐EV treatment attenuated the injury‐induced disruption of tight junction architecture and mucus layer integrity, whereas HDF‐EVs conferred minimal improvement. These observations were corroborated at the molecular level, as qPCR analysis showed reduced mRNA levels of *Tjp1*, *Ocln*, *Cldn1*, and *Muc2* after MPG injury, whereas western blotting confirmed corresponding reductions in ZO‐1, occludin, and claudin‐1 protein levels. These changes were partially attenuated by Sacral NC‐EV treatment (Figures 3H–K and S4C).

Given the close relationship between mucosal barrier disruption and apoptosis‐associated tissue injury, we further examined apoptosis‐related changes in the rectal mucosa. Western blot analysis of mucosa‐enriched rectal samples showed that MPG injury increased caspase‐3 and Bax levels and decreased Bcl‐2 levels (Figure 3L). Cleaved caspase‐3 immunofluorescence further showed increased staining after MPG injury that was visually concentrated within the anatomically defined epithelial region, whereas the signal was reduced following Sacral NC‐EV treatment (Figure S4D). Collectively, these findings indicate that MPG injury compromises rectal mucosal barrier integrity and increases apoptosis‐associated signaling in the rectal mucosa, whereas Sacral NC‐EV treatment attenuates both barrier disruption and apoptosis‐associated changes.

### 2.4 Sacral NC‐EVs Attenuate Structural and Vascular Alterations Following MPG Injury

To evaluate local pathological changes within the MPG after injury, we first examined tissue structure using Masson trichrome and toluidine blue staining. Masson trichrome staining and quantitative analysis showed increased collagen deposition and disrupted tissue architecture in the injured MPG. Both changes were partially attenuated by Sacral NC‐EV treatment but not by HDF‐EVs (Figures 4A and S5A). Toluidine blue staining further revealed loss of normal neuronal morphology and prominent vacuolar degeneration after MPG injury. Sacral NC‐EVs partially preserved neuronal architecture, whereas HDF‐EVs showed limited benefit (Figure 4B).

**FIGURE 4:**
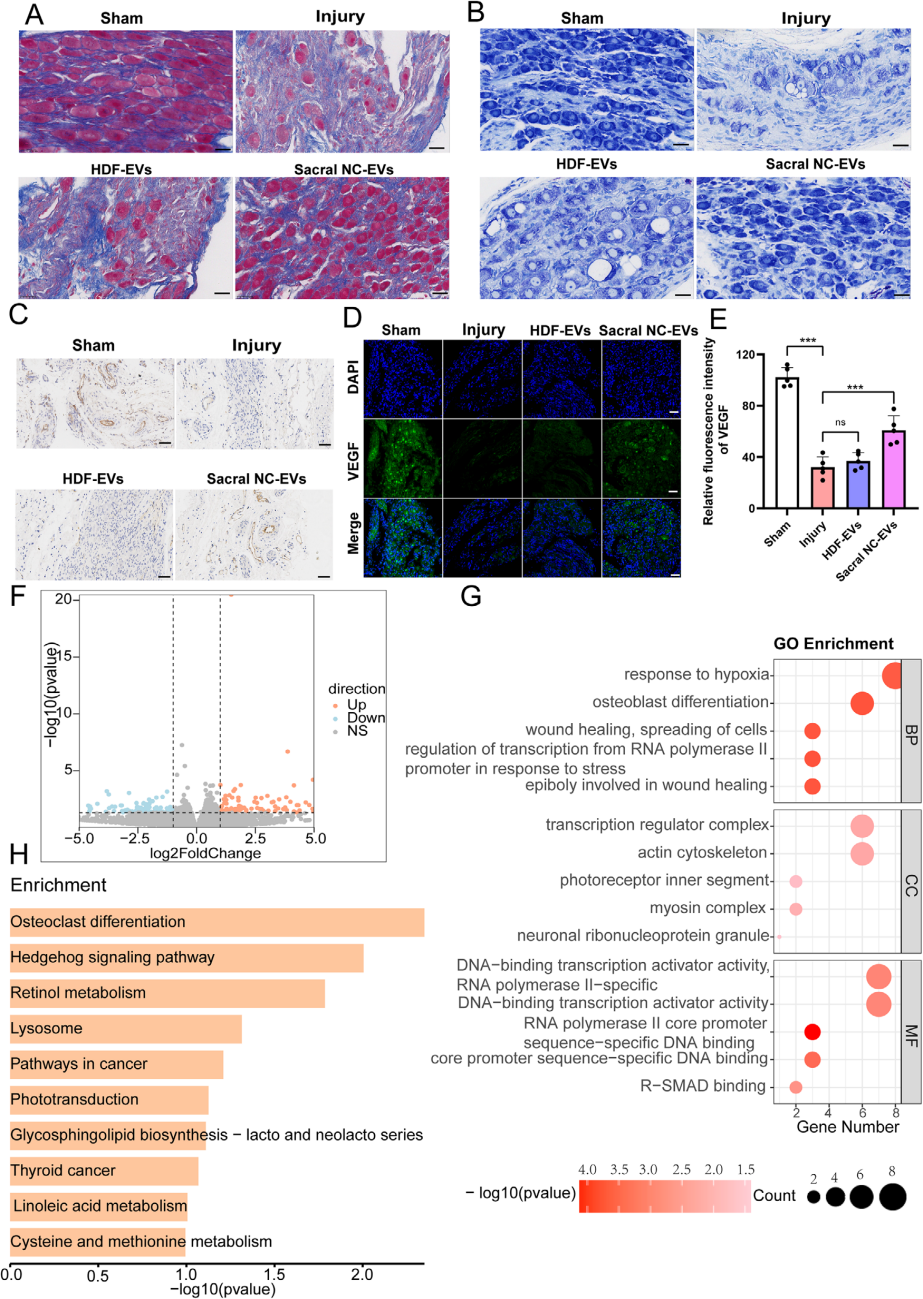
Sacral NC‐EVs attenuate structural damage and preserve vascular‐associated features following MPG injury. (A) Representative Masson trichrome staining of MPG sections from Sham, Injury, HDF‐EVs, and Sacral NC‐EVs groups. Scale bars, 25 µm. (B) Representative toluidine blue staining showing neuronal morphology in MPG tissues from each group. Scale bars, 25 µm. (C) Representative immunohistochemical staining of the endothelial marker CD31 in MPG tissues from each group. Scale bars, 50 µm. (D) Representative immunofluorescence staining of VEGF in MPG tissues from each group. Scale bars, 100 µm. (E) Quantification of relative VEGF fluorescence intensity; *n* = 5 rats per group. (F) Volcano plot showing differentially expressed genes between Sham and Injury MPG tissues; *n* = 3 biological replicates per group. (G,H) GO enrichment analysis (G) and KEGG pathway enrichment analysis (H) of genes upregulated in the Injury group relative to the Sham group. For panel E, data are presented as mean ± SD, and each dot represents one independent animal. *p* values for VEGF quantification were calculated using one‐way ANOVA followed by Tukey's multiple‐comparison test. ns, not significant; ^***^ *p *< 0.001.

The marked structural disruption observed in the injured MPG prompted us to examine whether it was accompanied by alterations in the local vascular compartment. Accordingly, CD31‐positive microvascular structures and VEGF expression were assessed as vascular‐associated readouts. At day 5 after injury, immunohistochemical staining showed reduced CD31‐positive microvascular structures after MPG injury, whereas Sacral NC‐EV treatment partially increased CD31 staining compared with injured controls (Figure 4C). Consistently, VEGF immunofluorescence was decreased in the injured MPG and was partially increased after Sacral NC‐EV treatment, while HDF‐EVs showed no comparable effect (Figure 4D,E).

To further define the molecular alterations at the same day‐5 time point, bulk RNA sequencing was performed on MPG tissues from the Sham and Injury groups. Using a *p* value < 0.05 and |log_2_FC| > 1, 203 differentially expressed genes were identified, including 90 genes expressed at higher levels in the Injury group and 113 expressed at higher levels in the Sham group, as illustrated by the volcano plot (Figure 4F). Functional enrichment analysis of the upregulated genes identified GO terms related to response to hypoxia, wound healing, stress‐responsive transcriptional regulation, and cytoskeletal organization (Figure 4G). KEGG analysis further identified pathways involving Hedgehog signaling, lysosomal function, and lipid and amino acid metabolism (Figure 4H). Together, these results indicate that genes upregulated after MPG injury are enriched in processes related to stress responses, transcriptional regulation, cytoskeletal organization, and metabolism.

Collectively, MPG injury produced early structural and vascular alterations accompanied by broad transcriptional changes, while Sacral NC‐EVs partially preserved tissue architecture and vascular‐associated features.

### 2.5 Sacral NC‐EVs Promote Recovery of Neuronal Architecture and Synaptic Plasticity‐Associated Signaling in the Injured MPG

To further characterize the transcriptional alterations induced by Sacral NC‐EVs in the injured MPG, transcriptomic sequencing was performed on MPG tissues from the Injury and Sacral NC‐EVs groups. Differentially expressed genes between the two groups are presented in the volcano plot (Figure 5A). GO enrichment analysis indicated that upregulated genes were primarily associated with synaptic function and neurotransmitter‐related processes, including neurotransmitter transport and synaptic signaling. Cellular component and molecular function terms further highlighted postsynaptic structures and ion channel activity (Figure 5B). KEGG analysis revealed enrichment in neuroactive ligand–receptor interaction, calcium signaling, and MAPK pathways (Figure 5C). These enrichment patterns indicate that Sacral NC‐EV treatment is associated with transcriptional changes related to synaptic signaling and activity‐dependent neuronal plasticity.

**FIGURE 5:**
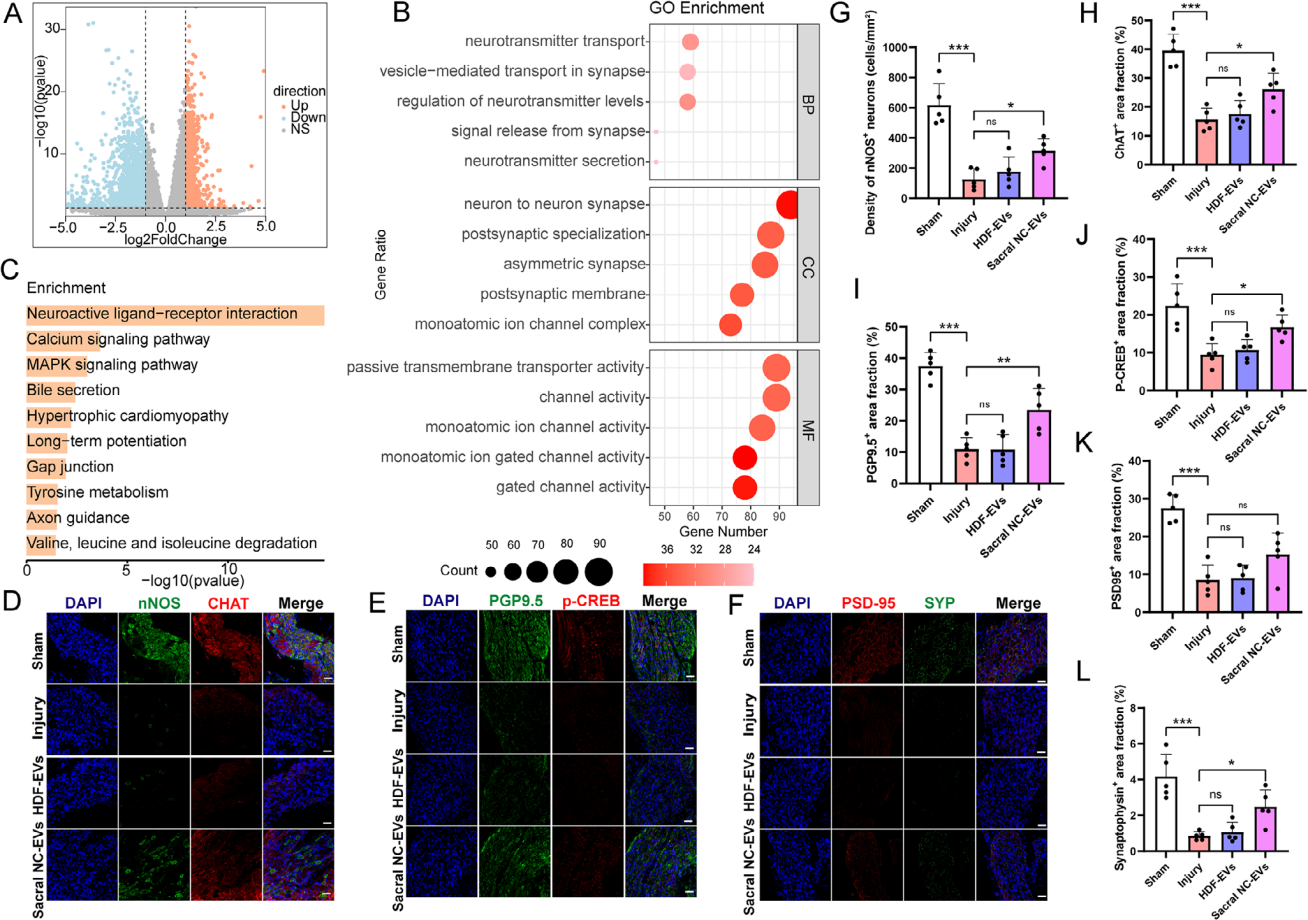
Sacral NC‐EVs promote recovery of neuronal architecture and synaptic plasticity‐associated signaling in the injured MPG. (A) Volcano plot showing differentially expressed genes between Injury and Sacral NC‐EVs groups in MPG tissues; *n* = 3 biological replicates per group. (B) GO enrichment analysis of upregulated genes in the Sacral NC‐EVs group. (C) KEGG pathway enrichment analysis of upregulated genes in the Sacral NC‐EVs group. (D–F) Representative immunofluorescence staining of nNOS and ChAT (D), PGP9.5 and p‐CREB (E), and PSD‐95 and synaptophysin (SYP) (F) in MPG sections from Sham, Injury, HDF‐EVs, and Sacral NC‐EVs groups. Scale bars, 50 µm. (G) Quantification of nNOS^+^ neuron density in MPG tissues. (H–L) Quantification of ChAT^+^ area fraction (H), PGP9.5^+^ area fraction (I), p‐CREB^+^ area fraction (J), PSD‐95^+^ area fraction (K), and SYP/synaptophysin^+^ area fraction (L) in MPG tissues; *n* = 5 rats per group for G–L. For panels G–L, data are presented as mean ± SD, and each dot represents one independent animal. *p* values for immunofluorescence quantification were calculated using one‐way ANOVA followed by Tukey's multiple‐comparison test. ns, not significant; ^*^ *p *< 0.05, ^**^ *p *< 0.01, ^***^ *p *< 0.001.

To determine whether these transcriptional changes were accompanied by structural neural recovery, we examined neuronal markers within the MPG. Immunofluorescence analysis showed that MPG injury markedly reduced nNOS^+^ neuronal density, ChAT^+^ area fraction, and PGP9.5^+^ area fraction, indicating impaired nitrergic, cholinergic, and pan‐neuronal structures within the MPG (Figure 5D,E,G–I). Sacral NC‐EV treatment significantly increased nNOS^+^ neuronal density as well as ChAT^+^ and PGP9.5^+^ area fractions compared with injured controls, whereas HDF‐EVs showed no significant improvement under the same conditions (Figure 5D,E,G–I).

We further assessed markers associated with neuronal activity and synaptic remodeling. MPG injury significantly decreased p‐CREB^+^ area fraction, reflecting reduced activation of CREB signaling involved in neuronal survival and activity‐dependent plasticity, and also reduced the synapse‐associated markers PSD‐95 and synaptophysin (SYP) (Figure 5E,F,J–L). Sacral NC‐EV treatment significantly increased p‐CREB^+^ and SYP^+^ area fractions compared with injured controls, whereas PSD‐95^+^ area fraction showed a non‐significant upward trend after treatment (Figure 5E,F,J–L). HDF‐EVs produced limited effects on these markers. Collectively, these findings suggest that Sacral NC‐EVs preserve neuronal structures within the injured MPG and enhance markers associated with neuronal activity and synaptic remodeling.

### 2.6 Sacral NC‐EVs Exhibit Distinct Effects on the Oxidative Stress–Pyroptosis Axis and Neuronal Recovery Compared With Other EV Sources

Given the structural, vascular, and transcriptomic alterations observed after MPG injury, together with prior evidence implicating oxidative stress and inflammasome signaling in neural injury, we next examined oxidative stress and pyroptosis‐associated signaling and assessed their modulation by Sacral NC‐EVs [32]. DHE staining revealed prominent ROS accumulation after MPG injury, accompanied by increased 8‐OHdG^+^ and NOX2^+^ areas, indicating enhanced oxidative damage and ROS‐generating activity (Figures 6A,B and S6A–D). Sacral NC‐EV treatment markedly reduced these oxidative stress‐associated changes, whereas HDF‐EVs showed limited effects (Figures 6A,B and S6A–D). In parallel, MPG injury increased GSDMD^+^ area fraction and enhanced NLRP3 and GSDMD staining, together with upregulation of pyroptosis‐related genes, including *Nlrp3*, *Casp1*, *Gsdmd*, *Il18*, and *Il1b* (Figures 6C–E and S6E–J). To examine the cellular distribution of the GSDMD response within the MPG, GSDMD was separately co‐stained with PGP9.5, S100β, and CD68. In injured MPG tissues, GSDMD immunoreactivity was associated with PGP9.5‐positive neuronal somata and partially overlapped with S100β‐positive glial and CD68‐positive macrophage profiles (Figure S6K,M). Because PGP9.5 clearly delineated neuronal somata, the neuronal GSDMD response was further quantified, showing an increased proportion of GSDMD‐immunoreactive PGP9.5‐positive somata after injury that was reduced by Sacral NC‐EV treatment (Figure S6K,L). These findings support neuronal involvement in the tissue‐level GSDMD response while suggesting that non‐neuronal populations may also contribute.

**FIGURE 6:**
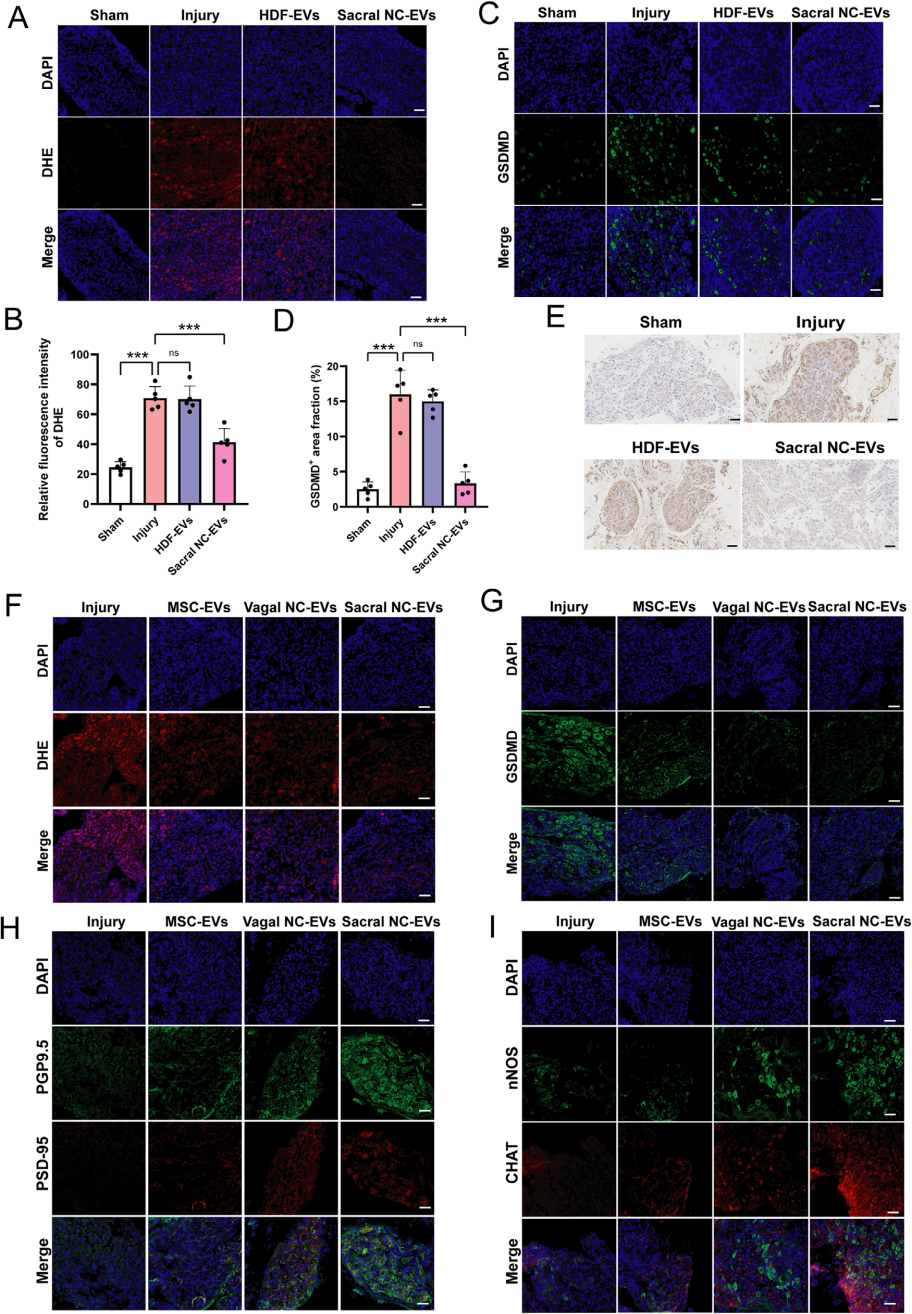
Sacral NC‐EVs exhibit distinct effects on the oxidative stress–pyroptosis axis and neuronal recovery compared with other EV sources. (A) Representative DHE staining images showing ROS levels in MPG tissues from Sham, Injury, HDF‐EVs, and Sacral NC‐EVs groups. Scale bars, 50 µm. (B) Quantification of relative DHE fluorescence intensity; *n* = 5 rats per group. (C) Representative immunofluorescence staining of GSDMD in MPG tissues from each group. Scale bars, 50 µm. (D) Quantification of GSDMD^+^ area fraction; *n* = 5 rats per group. (E) Representative immunohistochemical staining of NLRP3 in MPG tissues from each group. Scale bars, 50 µm. (F,G) Representative immunofluorescence staining of DHE (F) and GSDMD (G) in MPG tissues from Injury, MSC‐EVs, Vagal NC‐EVs, and Sacral NC‐EVs groups. Scale bars, 50 µm. (H) Representative immunofluorescence staining of PGP9.5 and PSD‐95 in MPG tissues from the indicated groups. Scale bars, 50 µm. (I) Representative immunofluorescence staining of nNOS and ChAT in MPG tissues from the indicated groups. Scale bars, 50 µm. Corresponding quantification of panels F–I is shown in Figure S8F–K; *n* = 3 rats per group. Data are presented as mean ± SD for quantitative panels. Each dot represents one independent animal. *P* values were calculated using one‐way ANOVA followed by Tukey's multiple‐comparison test. ns, not significant; ^*^ *p *< 0.05, ^***^ *p *< 0.001.

Building on this evidence of neuronal involvement in vivo, we next used differentiated PC‐12 cells as a controlled neuronal‐like model to examine whether Sacral NC‐EVs could attenuate oxidative stress and pyroptosis‐associated changes induced by H_2_O_2_. H_2_O_2_ exposure induced robust ROS accumulation and activated pyroptosis‐related signaling, as evidenced by increased NLRP3, GSDMD‐N, and cleaved caspase‐1 levels (Figure S7A–C). Consistent with these molecular changes, H_2_O_2_ also promoted IL‐1β and IL‐18 secretion and increased LDH release, indicating inflammatory cytokine release and membrane damage (Figure S7D–F). Sacral NC‐EV treatment reduced ROS accumulation, suppressed pyroptosis‐related protein changes, and attenuated cytokine release and LDH leakage (Figure S7A–F). These in vitro findings further support the protective effect of Sacral NC‐EVs against oxidative stress‐induced pyroptotic injury.

To determine whether the protective profile observed with Sacral NC‐EVs differed from that of other EV preparations, we next evaluated MSC‐EVs and Vagal NC‐EVs under the same experimental conditions. MSC‐EVs were included as a widely studied therapeutic EV comparator with recognized anti‐inflammatory and reparative potential, whereas Vagal NC‐EVs served as a neural crest–derived comparator with a distinct axial identity from Sacral NC‐EVs. Before functional comparison, Vagal NC identity was confirmed by expression of the neural crest markers SOX10 and NGFR, together with immunofluorescence detection of HOXB4 and qPCR evidence of *HOXB3* and *HOXB5* induction (Figure S8A–C). Western blotting confirmed enrichment of the EV markers CD9, CD81, and TSG101 and the absence of CALNEXIN in MSC‐EVs and Vagal NC‐EVs. Comparative analysis further revealed source‐associated protein patterns: CD73 was more prominent in MSC‐EVs, Vimentin and FN1 were more prominent in HDF‐EVs, and NGFR was preferentially detected in Vagal and Sacral NC‐EVs (Figure S8D,E).

In the injured MPG, all three EV treatments attenuated oxidative stress‐ and pyroptosis‐associated changes relative to the Injury group, with Sacral NC‐EVs producing the greatest reductions in DHE fluorescence and GSDMD^+^ area fraction (Figures 6F,G and S8F,G). Sacral NC‐EVs also produced the greatest increases in PGP9.5^+^ and ChAT^+^ area fractions. PSD‐95^+^ area fraction and nNOS^+^ neuronal density were numerically highest in the Sacral NC‐EV group, but the differences relative to the Vagal NC‐EV group did not reach statistical significance (Figures 6H,I and S8H–K). Collectively, these comparative findings support the therapeutic potential of EVs derived from Sacral NC, an embryonic progenitor population that contributes to pelvic autonomic ganglion development, for MPG repair.

### 2.7 Sacral NC‐EV‐Associated ANXA1 Contributes to the Suppression of Oxidative Stress and Pyroptosis via FPR2 Signaling

Having identified oxidative stress and pyroptosis‐associated signaling as prominent features of MPG injury that were attenuated by Sacral NC‐EVs, we next sought to identify EV‐associated proteins that might contribute to these effects. DIA‐based proteomic profiling was performed on Sacral NC‐EVs and HDF‐EVs, using HDF‐EVs as a differentiated, non‐neural somatic EV reference. Three independent biological replicates were analyzed per group. Across all samples, 2877 proteins were identified, with peptide‐ and protein‐level false discovery rates controlled at <1%. Among the identified proteins, 2590 (90.0%) overlapped with ExoCarta human EV protein annotations, including 94 of the ExoCarta Top 100 EV proteins, while 2652 (92.2%) overlapped with Vesiclepedia human protein annotations. These findings support the EV‐associated nature of the proteomic dataset.

GO enrichment analysis showed that proteins enriched in Sacral NC‐EVs were associated with biological processes relevant to the observed therapeutic phenotype, including neurogenesis, oxidative stress regulation, angiogenesis, axon growth, and pyroptosis‐related signaling (Figure 7A–D). Differential protein abundance between Sacral NC‐EVs and HDF‐EVs is shown in the volcano plot (Figure 7E). Sequential filtering based on differential abundance and consistency across biological replicates retained 235 reproducibly enriched candidates. Among these candidates, ANXA1 was prioritized based on its marked enrichment (log_2_FC = 6.045; *p* = 1.74 × 10^−^^4^) and reported relevance to inflammasome‐mediated pyroptosis. Western blotting further showed greater ANXA1 abundance in Sacral NC‐EVs than in HDF‐EVs, MSC‐EVs, or Vagal NC‐EVs, whereas CD81 was detected across all EV preparations (Figure S9A). These findings supported the prioritization of ANXA1 for functional investigation.

**FIGURE 7:**
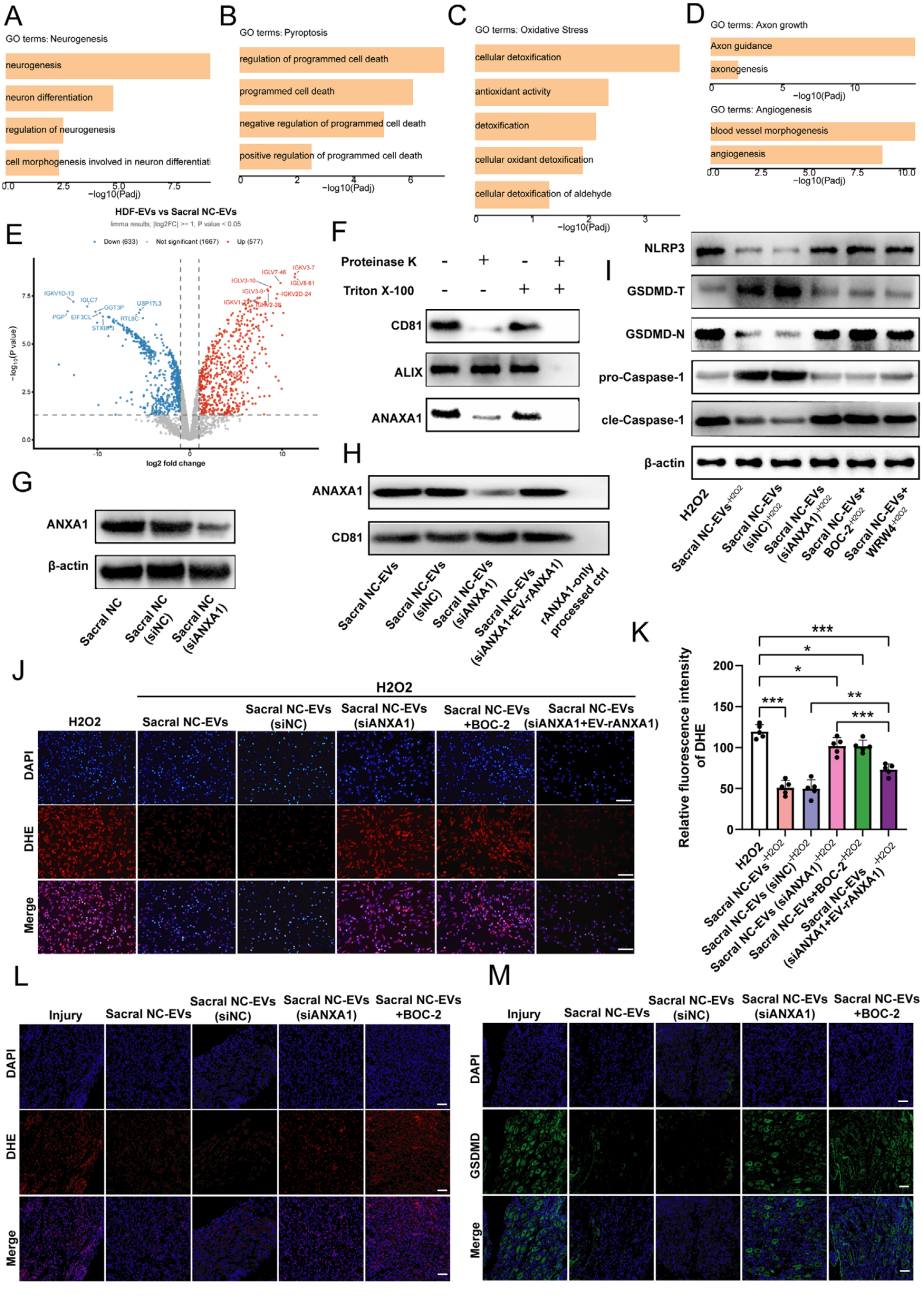
Sacral NC‐EV‐associated ANXA1 contributes to the suppression of oxidative stress and pyroptosis via FPR2 signaling. (A–D) GO enrichment analysis of proteins enriched in Sacral NC‐EVs compared with HDF‐EVs, showing enrichment in biological processes related to neurogenesis (A), pyroptosis (B), oxidative stress (C), and axon growth and angiogenesis (D). (E) Volcano plot showing differentially abundant proteins between Sacral NC‐EVs and HDF‐EVs. Proteomic profiling was performed using three independent EV preparations per group. (F) Proteinase K protection assay of Sacral NC‐EVs in the absence or presence of Triton X‐100, showing the accessibility of EV‐associated ANXA1 to protease digestion. CD81, ALIX, and ANXA1 were detected by western blot. (G) Western blot analysis of ANXA1 expression in Sacral NC after siRNA‐mediated *ANXA1* knockdown, with siNC as a negative control. (H) Western blot analysis of ANXA1 depletion and reconstitution in Sacral NC‐EVs. siANXA1 treatment reduced ANXA1 abundance in Sacral NC‐EVs, whereas incubation with recombinant ANXA1 restored ANXA1 detection in washed siANXA1 Sacral NC‐EVs. A recombinant ANXA1‐only processed control was prepared in parallel without EVs. (I) Western blot analysis of pyroptosis‐related proteins, including NLRP3, GSDMD‐T, GSDMD‐N, pro‐caspase‐1, and cleaved caspase‐1, in H_2_O_2_‐treated PC‐12 cells under the indicated treatments. (J) Representative DHE staining images showing ROS levels in H_2_O_2_‐treated PC‐12 cells under the indicated treatments. Scale bars, 100 µm. (K) Quantification of relative DHE fluorescence intensity in PC‐12 cells; *n* = 5 independent experiments. (L,M) Representative DHE staining (L) and GSDMD immunofluorescence staining (M) in MPG tissues from the indicated treatment groups; *n* = 3 rats per group. Corresponding quantification is shown in Figure S9G,H. Scale bars, 50 µm. Data are presented as mean ± SD for quantitative panels. *p* values were calculated using one‐way ANOVA followed by Tukey's multiple‐comparison test. ns, not significant; ^*^ *p *< 0.05, ^**^ *p *< 0.01, ^***^ *p *< 0.001.

We next examined how ANXA1 was associated with Sacral NC‐EVs. Proteinase K treatment markedly reduced ANXA1 abundance in intact EVs, with further digestion following Triton X‐100‐mediated membrane permeabilization, indicating that a substantial proportion of EV‐associated ANXA1 was externally accessible (Figure 7F). ANXA1 was retained after high‐salt washing and EV re‐isolation, supporting its stable association with Sacral NC‐EVs rather than loose adsorption of soluble protein (Figure S9B). Together, these findings support the presence of a stably EV‐associated and externally accessible ANXA1 fraction compatible with receptor‐mediated signaling.

To establish ANXA1 loss‐of‐function and reconstitution models, ANXA1 was depleted in donor Sacral NC using siRNA. qPCR and western blotting confirmed efficient knockdown in siANXA1‐treated cells, with a corresponding reduction in ANXA1 abundance in the derived EVs, whereas siNC treatment had no appreciable effect (Figures 7G,H and S9C). To optimize ANXA1 reconstitution, siANXA1 Sacral NC‐EVs were incubated with 0.1, 0.25, or 0.5 µg of recombinant ANXA1 per 1 × 10^10^ EV particles, followed by ultracentrifugation and PBS washing. The post‐wash ANXA1 signal increased across the tested inputs and was highest at 0.5 µg per 1 × 10^10^ EV particles; this condition was therefore selected for subsequent experiments (Figure S9D). Western blotting confirmed ANXA1 detection in the washed, reconstituted EV preparation, hereafter referred to as EV‐rANXA1. Only a minimal ANXA1 signal was detected in the EV‐free rANXA1 control processed in parallel, arguing against substantial nonspecific pelleting of free recombinant protein (Figure 7H).

Given that ANXA1 has been reported to signal through FPR2 [33, 34, 35], we next examined the functional contribution of ANXA1 and its dependence on FPR2 in H_2_O_2_‐treated PC‐12 cells. Sacral NC‐EVs and siNC Sacral NC‐EVs reduced NLRP3, GSDMD‐N, and cleaved caspase‐1 levels, whereas ANXA1 depletion or FPR2 blockade attenuated these effects (Figure 7I). Consistently, ANXA1 depletion and FPR2 blockade with BOC‐2 or WRW4 weakened Sacral NC‐EV‐mediated suppression of DHE fluorescence, whereas reconstitution of siANXA1 Sacral NC‐EVs with recombinant ANXA1 partially restored their antioxidant activity (Figures 7J,K and S9E,F). To assess whether EV association influenced ANXA1 activity, free recombinant ANXA1 was added directly to H_2_O_2_‐treated cells receiving siANXA1 Sacral NC‐EVs. Free ANXA1 modestly reduced ROS, but its effect did not increase further between 4 and 6 µg/mL and remained weaker than that of EV‐rANXA1 (Figure S9E,F), suggesting that EV association may enhance the antioxidant activity of ANXA1.

We further examined whether ANXA1–FPR2 signaling contributed to Sacral NC‐EV‐mediated protection in vivo. In injured MPG tissues, Sacral NC‐EVs and siNC Sacral NC‐EVs comparably reduced DHE fluorescence and GSDMD^+^ area fraction, whereas these effects were attenuated by ANXA1 depletion or FPR2 blockade with BOC‐2 (Figures 7L,M and S9G,H). Similarly, Sacral NC‐EVs and siNC Sacral NC‐EVs reduced 8‐OHdG^+^ and NOX2^+^ area fractions, whereas ANXA1 depletion or FPR2 blockade weakened these reductions (Figure S9I–L). Together, these in vitro and in vivo findings indicate that Sacral NC‐EV‐associated ANXA1 contributes to the suppression of oxidative stress and pyroptosis‐associated signaling through FPR2, while the residual protective activity observed after ANXA1 depletion suggests that additional EV cargo may also contribute to the overall response.

## 3 Discussion

Pelvic autonomic nerve injury is a clinically important complication of pelvic surgery and can lead to persistent postoperative bowel dysfunction, for which effective therapies remain limited [2]. Here, we demonstrate that Sacral NC‐EVs promote repair of the injured MPG by alleviating oxidative stress and inhibiting pyroptosis. Improved MPG integrity was accompanied by partial recovery of rectal neural innervation and preservation of rectal mucosal barrier integrity. Comparisons across EV sources showed that Sacral NC‐EVs more effectively attenuated changes associated with oxidative stress and pyroptosis and increased PGP9.5‐positive and ChAT‐positive neuronal areas within the injured MPG than MSC‐EVs and Vagal NC‐EVs. Integrated proteomic and functional analyses further support EV‐associated ANXA1 as a contributor to these protective effects through FPR2 signaling. Together, these findings support Sacral NC‐EVs as a promising cell‐free strategy for pelvic nerve repair (Figure 8).

**FIGURE 8:**
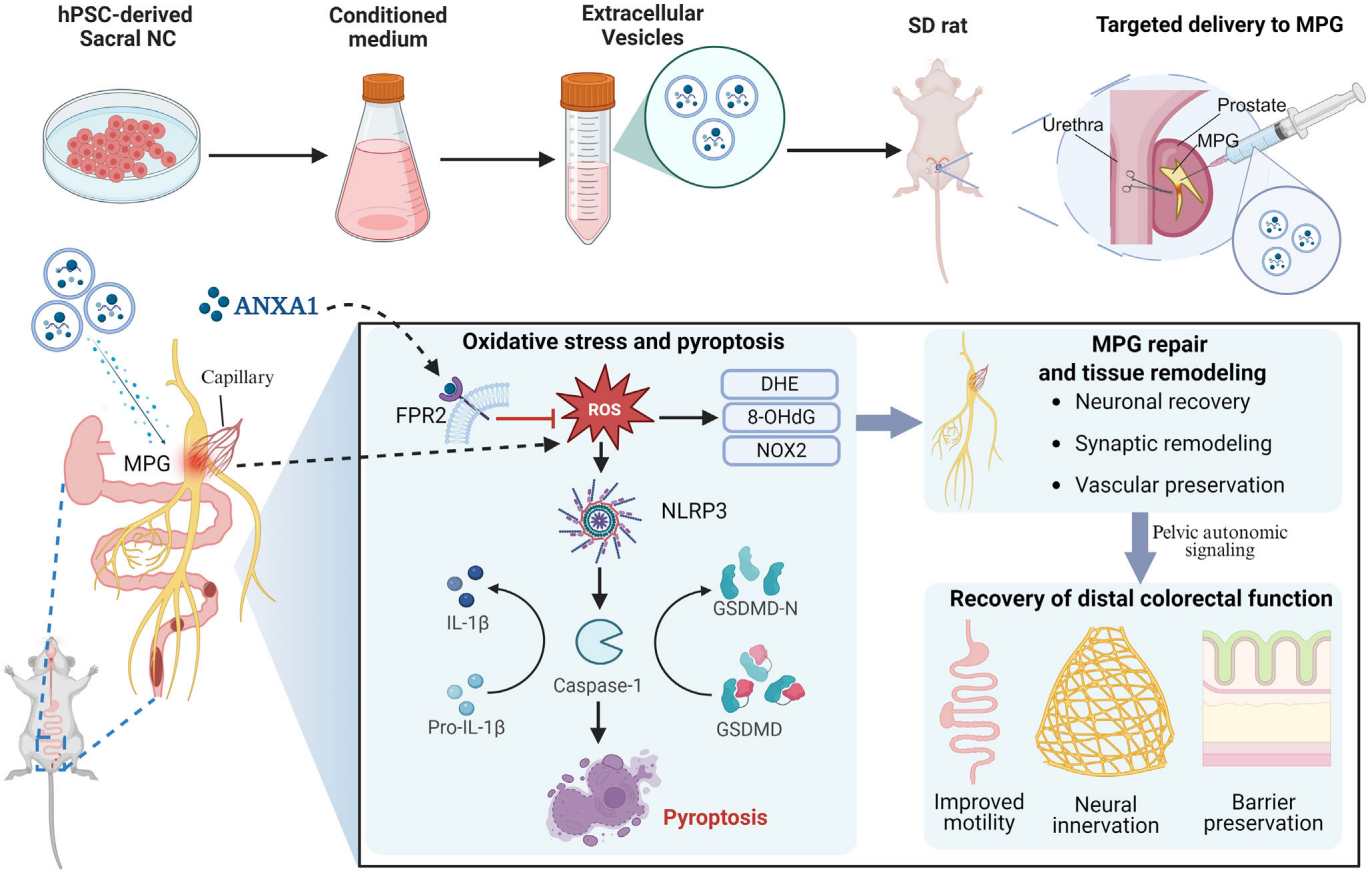
Sacral NC‐EVs improve colorectal function by repairing the injured MPG. Schematic illustration showing that MPG injury induces oxidative stress‐associated pyroptotic signaling and disrupts neural regulation of the colorectum. Sacral NC‐EVs attenuate oxidative stress and pyroptosis, at least in part through ANXA1–FPR2 signaling, thereby promoting MPG neuroregeneration and microenvironmental remodeling. Improved MPG integrity contributes to recovery of distal colorectal function, including improved motility, ENS reinnervation, and barrier integrity.

The MPG functions as a central integration hub of pelvic autonomic signaling, coordinating the innervation of multiple pelvic organs, including the distal colorectum, bladder, and reproductive organs [1]. Consequently, injury to the MPG can result in widespread pelvic organ dysfunction. Clinically, intestinal dysfunction is a common consequence of pelvic autonomic nerve injury following pelvic surgery; however, its manifestations are heterogeneous, ranging from fecal urgency and incontinence to impaired colonic motility and constipation [4, 36, 37]. These diverse phenotypes likely reflect differences in the anatomical level and severity of neural injury [4, 38, 39]. In the present study, the injury was localized to the MPG and predominantly produced constipation‐like symptoms, consistent with impaired pelvic autonomic regulation of propulsive colorectal motility.

Although numerous studies have demonstrated that pelvic autonomic nerve injury leads to intestinal dysfunction, the structural changes in the intestinal neural network following injury remain poorly characterized [40]. Here, we demonstrate that MPG injury induces marked reductions in TUJ1‐positive nerve fibers and NeuN‐positive neurons within the rectal myenteric plexus, indicating secondary degeneration of the ENS. Although the ENS is often regarded as a relatively autonomous neural network capable of independently regulating gastrointestinal function, accumulating evidence indicates that its activity is continuously modulated by extrinsic autonomic inputs [41, 42]. Postganglionic autonomic projections arising from the MPG constitute an important component of the extrinsic innervation of the distal colorectum [7]. Upon entering the intestinal wall, these fibers project to the myenteric and submucosal plexuses, where they establish synaptic contacts with enteric neurons and regulate neural circuits controlling intestinal motility and mucosal function [6, 43].

The reduction of ENS neuronal markers observed after MPG injury likely represents an important structural basis for post‐injury intestinal dysfunction. This phenomenon may arise from two potentially interconnected mechanisms. First, disruption of pelvic autonomic projections from the MPG to the intestinal wall may contribute to secondary degeneration of enteric neurons, potentially by reducing the neurotrophic support provided by extrinsic autonomic innervation [44, 45, 46]. Second, MPG injury‐induced intestinal dysmotility leads to fecal retention and sustained elevation of intraluminal pressure within the distal intestine, potentially damaging the intestinal epithelium and compromising mucosal barrier integrity. In addition, MPG injury‐induced impairment of the ENS may further compromise intestinal barrier function [47, 48, 49, 50]. Barrier disruption permits luminal microbes and inflammatory mediators to penetrate the intestinal wall, triggering local inflammation that may further injure intramural nerves [51, 52]. These processes could establish a self‐perpetuating pathological cycle in which neural injury exacerbates intestinal dysfunction, which in turn accelerates enteric neuronal damage.

Previous studies in both the central and peripheral nervous systems have established that cellular oxidative stress and pyroptosis critically impede neural regeneration following injury [10, 53]. Motivated by these findings, we focused on the canonical pyroptotic pathway, which is predominantly activated by endogenous DAMPs in sterile nerve injury, whereas noncanonical pathways are more commonly associated with the cytosolic sensing of bacterial LPS [54, 55, 56]. In this pathway, nerve injury disrupts mitochondrial function and activates NADPH oxidase, leading to excessive ROS production, NLRP3 inflammasome activation, caspase‐1‐mediated GSDMD cleavage, and subsequent release of pro‐inflammatory cytokines including IL‐1β and IL‐18 [55, 57, 58]. In our study, MPG injury induced oxidative stress and was accompanied by increased canonical pyroptosis‐associated signaling. Notably, treatment with Sacral NC‐EVs reversed these changes, suggesting that their neuroprotective effects are mediated through suppression of oxidative stress and pyroptosis, thereby establishing a microenvironment favorable for neural repair.

The contribution of Sacral NC to the embryonic development of pelvic autonomic ganglia provided the biological rationale for selecting Sacral NC as an EV donor source for MPG repair. Compared with MSC‐EVs and Vagal NC‐EVs, Sacral NC‐EVs produced greater reductions in oxidative stress and pyroptosis‐associated changes and greater restoration of PGP9.5^+^ and ChAT^+^ neuronal areas, although differences in PSD‐95 and nNOS measures relative to Vagal NC‐EVs did not reach statistical significance. These differences were unlikely to reflect EV input alone, because all preparations were administered at the same nominal protein dose and corresponded to a broadly comparable estimated particle‐dose range. Moreover, HDF‐EVs and Sacral NC‐EVs showed similar tissue‐level localization within PGP9.5^+^ regions of the injured MPG, indicating that their different therapeutic effects could not be readily explained by gross local distribution. The therapeutic profile of Sacral NC‐EVs may therefore reflect source‐dependent differences in EV cargo. However, whether the HOX‐defined positional identity of the donor cells is reflected in EV cargo, or whether cell‐type‐specific uptake or receptor engagement contributes to the observed differences, remains to be determined [15, 16]. Together, these findings support Sacral NC‐EVs as a promising candidate for MPG repair and highlight the developmental relationship between Sacral NC and pelvic autonomic ganglia as a biologically relevant basis for EV donor‐source selection.

Integrated proteomic and functional analyses support EV‐associated ANXA1 as one contributor to the protective effects of Sacral NC‐EVs. Loss‐of‐function, reconstitution, and FPR2 blockade experiments collectively indicate that these effects are compatible with ANXA1–FPR2 signaling. The stable and surface‐accessible association of ANXA1 with Sacral NC‐EVs provides a plausible basis for its engagement with cell‐surface FPR2 [59, 60]. Notably, EV‐rANXA1 showed greater antioxidant activity than free ANXA1, suggesting that EV association may enhance ANXA1 stability, local presentation, or receptor engagement, although the underlying mechanism remains to be determined. Previous evidence that ANXA1‐bearing EVs activate FPR‐dependent reparative signaling further supports the biological activity of EV‐associated ANXA1 [61, 62]. The activity of human ANXA1 in the rat model further supports the cross‐species conservation of ANXA1–FPR2 signaling [63, 64]. Because ANXA1 depletion or FPR2 blockade only partially attenuated the protective effects, other enriched proteins and EV‐associated non‐coding RNAs may act independently or cooperatively with ANXA1 to support MPG repair [65].

Given the ability of Sacral NC‐EVs to attenuate oxidative stress and pyroptosis after MPG injury, future studies may explore strategies to further enhance their therapeutic efficacy, such as optimizing EV delivery or engineering EV cargo to strengthen their neuroprotective properties [66]. Several limitations should be acknowledged. First, although the functional experiments support EV‐associated ANXA1 as a contributor to the protective effects of Sacral NC‐EVs, other enriched proteins and non‐coding RNAs may act independently or cooperatively with ANXA1. Second, GSDMD co‐staining supported neuronal involvement and suggested possible glial and macrophage involvement but did not resolve the relative contributions of these populations. Moreover, differentiated PC‐12 cells do not fully recapitulate primary pelvic autonomic neurons or the multicellular MPG microenvironment, warranting further cell‐type‐resolved analyses. Third, although PKH26 tracing showed the spatial association of Sacral NC‐EVs with neuronal and glial cells, it did not quantify cell‐type‐specific uptake or confirm intracellular cargo delivery. Fourth, the in vivo experiments evaluated a single EV dose and included only male rats. Although EV administration was standardized by total protein rather than particle number, the NTA‐derived particle inputs were broadly comparable among the EV formulations. Nevertheless, precise dose equivalence, the minimum effective dose, the dose–response relationship, the therapeutic window, and applicability to females remain to be established. Fifth, the absence of a formal a priori power calculation may have limited the precision of effect estimates and the ability to detect modest differences in selected comparisons. Finally, human‐derived EVs were administered to immunocompetent rats without immunosuppression. Although previous studies suggest relatively low immunogenicity in this setting [67, 68], xenogeneic immune responses were not specifically assessed. The durability, safety, and immunogenicity of Sacral NC‐EV treatment therefore require evaluation in longer‐term and more clinically relevant models, together with further optimization of EV delivery and manufacturing consistency.

In summary, local administration of Sacral NC‐EVs improved MPG integrity and downstream colorectal outcomes after injury. EV‐associated ANXA1 contributed to these effects by attenuating oxidative stress and pyroptosis‐associated signaling, at least in part through FPR2 signaling. These findings support Sacral NC‐EVs, derived from an embryonic progenitor population contributing to pelvic autonomic ganglion development, as a promising cell‐free approach to pelvic autonomic nerve repair.

## 4 Experimental Section

### 4.1 Differentiation of hPSCs Into Sacral NC and Vagal NC

To generate Sacral NC, hPSCs were initially cultured in Essential 6 (E6) medium on Day 0. On Day 1, fibroblast growth factor 8b (FGF8b, 500 ng mL^−^^1^) was added to promote posterior fate acquisition. Cells were subsequently replated onto Matrigel‐coated dishes at a density of 1.5 × 10^5^ cells cm^−^^2^ and maintained in E6 medium supplemented with FGF8b (500 ng mL^−^^1^) and CHIR99021 (3 µM), with daily medium changes for two consecutive days. On Day 5, cells were transferred to freshly Matrigel‐coated plates and cultured in the presence of growth differentiation factor 11 (GDF11, 50 ng mL^−^^1^) to further enhance posterior axial patterning until Day 7. From Day 8 onward, cells were maintained in a defined basal differentiation medium containing SB431542 (2 µM), CHIR99021 (1 µM), DMH1 (1 µM), and BMP4 (15 ng mL^−^^1^). This differentiation protocol was continued until Day 15, after which CD271^+^/CD49d^+^ Sacral NC were isolated by fluorescence‐activated cell sorting for subsequent experiments [29]. Vagal neural crest cells were generated from hPSCs using a modified serum‐free monolayer differentiation protocol based on previously reported retinoic acid (RA)–mediated vagal/enteric neural crest specification strategies [69, 70]. Briefly, hPSCs were treated with CHIR99021 (2 µM), SB431542 (2 µM), and SAG (2 µM) for 4 days, followed by RA treatment (1 µM) from day 4 to day 10 to promote vagal axial identity. The resulting cells were sorted and used for EV isolation and functional assays. Vagal NC identity was confirmed by neural crest marker expression and vagal‐level HOX gene profiling.

### 4.2 Establishment of the Bilateral MPG Injury Model

Bilateral MPG injury was induced using a previously described clamp injury model [29, 71]. Briefly, rats were anesthetized with inhaled isoflurane and placed in the supine position on a heated surgical platform. After sterile preparation, a 4–5 cm lower midline abdominal incision was made to expose the pelvic cavity. The prostate gland was gently retracted to identify the bilateral MPG located posterolateral to the prostate. For the injury groups, each MPG was clamped using a non‐serrated micro‐hemostat to induce standardized mechanical injury. In sham‐operated animals, the MPG was exposed and gently manipulated but not clamped.

### 4.3 EV Administration

Immediately after MPG injury, EVs were locally administered to the peri‐ganglionic region by multipoint microinjection. Each MPG side received 10 µL of EV suspension formulated to a common nominal total‐protein input of approximately 10 µg, as determined by BCA assay. NTA characterization of the administration formulations yielded estimated particle inputs of approximately 8.6 × 10^10^ particles per MPG side for Sacral NC‐EVs, 9.7 × 10^10^ particles per MPG side for HDF‐EVs, 8.7 × 10^10^ particles per MPG side for MSC‐EVs, and 9.9 × 10^10^ particles per MPG side for Vagal NC‐EVs. Thus, the estimated particle inputs were within the same order of magnitude and differed by no more than approximately 15% across the four EV sources. EV dosing was standardized by nominal total‐protein input rather than particle number; the NTA‐derived particle counts were reported as complementary characterization of the administered formulations and were not used for dose normalization. The Injury group received an equal volume of sterile PBS. No immunosuppressive treatment was administered during the study.

### 4.4 ANXA1–FPR2 Pathway Inhibition In Vivo

To evaluate the contribution of ANXA1–FPR2 signaling to the therapeutic effects of Sacral NC‐EVs, the formyl peptide receptor antagonist BOC‐2 was administered in vivo. Rats in the Sacral NC‐EVs + BOC‐2 group received bilateral MPG injection of Sacral NC‐EVs as described above, followed by intraperitoneal injection of BOC‐2 (50 µg kg^−^^1^) immediately after surgery and once daily for four consecutive days.

### 4.5 Tissue Collection and Experimental Timeline

To investigate early molecular events after injury, a subset of animals was sacrificed on day 5 postinjury, and MPG tissues were harvested for analysis of local pathological alterations, including neural structure, vascular integrity, oxidative stress, inflammasome activation, and transcriptomic changes. For evaluation of long‐term functional recovery, the remaining animals were assessed on day 30 postinjury. Gastrointestinal motility tests were performed, followed by the collection of distal rectal tissues to analyze enteric neuronal integrity and intestinal barrier function.

### 4.6 Statistical Analysis

Group sizes were prespecified before data collection and were selected to balance the planned experimental objectives with the reduction principle of the 3Rs. A formal a priori power calculation was not performed. Data are presented as the mean ± standard deviation (SD), except for violin plots, which show the median and quartiles. Technical replicates from the same biological sample were averaged before analysis, and the exact biological sample size (n) is provided in the corresponding figure legends. For datasets subjected to parametric analysis, normality and homogeneity of variance were evaluated before statistical testing. Comparisons between two independent groups were performed using a two‐sided unpaired Student's *t*‐test or a two‐sided Mann–Whitney *U* test, as appropriate. Comparisons among three or more groups were performed using one‐way ANOVA followed by Tukey's multiple comparisons test. All statistical tests were two‐sided, and *p *< 0.05 was considered statistically significant. Statistical analyses were performed using GraphPad Prism.

### 4.7 Ethics Approval Statement

All animal experiments were approved by the Institutional Animal Care and Use Committee of Sun Yat‐sen University (approval ID: SYSU‐IACUC‐2024‐001528). The use of human specimens in this study was approved by the Ethics Committee of the First Affiliated Hospital of Sun Yat‐sen University [approval number (2021):045].

## Author Contributions

T.Y., W.W.L., W.Q.L., A.P.X., and W.S. conceived and designed the study. T.Y., W.W.L., and Y.W. performed the experiments with assistance from Y.M., H.W., X.W., T.L., Q.T., and Y.Z., T.Y., and W.W.L. analyzed the data and prepared the figures. T.Y., W.W.L., and W.S. drafted the manuscript. W.Q.L., A.P.X., and W.S. critically revised the manuscript. W.S. acquired funding. W.Q.L., A.P.X., and W.S. supervised the study. All authors read and approved the final version of the manuscript.

## Funding

This work was supported by the National Natural Science Foundation of China (82573282, 82070529), the Natural Science Foundation of Guangdong Province (2025A1515011210), and the Guangdong Provincial Engineering Research Center for Stem Cell Therapy of Intractable Digestive Diseases.

## Conflicts of Interest

The authors declare no conflicts of interest.

## Supporting information




**Supporting File**: advs78321‐sup‐0001‐SuppMat.docx.

## Data Availability

The RNA‐sequencing data generated in this study have been deposited in the NCBI Gene Expression Omnibus (GEO) under accession number GSE338995. The mass spectrometry proteomics data have been deposited with the ProteomeXchange Consortium via the iProX partner repository under dataset identifier PXD081233. All other data supporting the findings of this study are available in the main text and Supporting Information or from the corresponding author upon reasonable request.
