# Supplementary material for: Sacral Neural Crest‐Derived Extracellular Vesicles Promote Pelvic Ganglion Repair Through Anti‐Pyroptotic Effects Involving ANXA1–FPR2 Signaling
Source: Adv Sci (Weinh). 2026 Oct 15:e78321. Online ahead of print. doi: 10.1002/advs.78321 (PMC13672682; doi:10.1002/advs.78321)
Supplement: Supplementary file 1 [file ADVS-9999-e78321-s001.docx]

Supporting Information for

**Sacral Neural Crest–Derived Extracellular Vesicles Promote Pelvic Ganglion Repair through Anti-Pyroptotic Effects Involving ANXA1–FPR2 Signaling**

**Supplementary Materials**

**Experimental Section**

**Culture of hPSCs and human bone marrow–derived MSCs**

hPSCs and human bone marrow-derived MSCs used in this study were generously provided by Professor Andy Peng Xiang (Center for Stem Cell Biology and Tissue Engineering, Sun Yat-sen University)[1,2]. hPSCs were cultured on Matrigel-coated six-well plates pre-equilibrated at 37 °C and maintained in Essential 8 (E8) medium under standard conditions (37 °C, 5% CO₂). Cells were passaged at 70–85% confluence using ReLeSR™ (STEMCELL Technologies) and routinely maintained at appropriate split ratios to preserve pluripotency. MSCs were maintained as previously described, passaged with trypsin–EDTA at approximately 80% confluence, and used between passages 5 and 8[3].

**Human dermal fibroblasts (HDFs)**

Human dermal fibroblasts (HDFs) were obtained from iCell Bioscience Inc. (Shanghai, China) and cultured in Primary Fibroblast Culture System (iCell Bioscience) according to the manufacturer's instructions. Cells were maintained at 37 °C in a humidified atmosphere containing 5% CO₂ and used between passages 3 and 8 for all experiments. For EV collection, HDFs were cultured to 70–80% confluence, washed twice with sterile phosphate-buffered saline (PBS), and then cultured in serum-free medium for 36 h. The conditioned medium was collected and subjected to EV isolation as described below.

**Human tissue samples**

Human rectal muscularis propria specimens were obtained from patients undergoing surgery at the Department of Gastrointestinal Surgery, the First Affiliated Hospital of Sun Yat-sen University. Tissues were collected from grossly normal areas ≥5 cm from the tumor margin and confirmed tumor-free by pathology. Patients were grouped based on pelvic surgery history: Control group (no prior pelvic surgery) and Injury group (prior pelvic surgery involving pelvic autonomic nerve regions, including radical prostatectomy, rectal cancer resection, or gynecologic malignancy resection). Inclusion criteria: age 40**–**70 years, elective surgery, and for Injury group, an interval ≥6 months between surgeries. Exclusion criteria: pelvic radiotherapy, inflammatory bowel disease, diabetic neuropathy, neurological disorders, medications affecting gut motility, tumor invasion of pelvic nerves, or severe infections/autoimmune diseases. All participants provided written informed consent before tissue collection. The use of human specimens was approved by the Ethics Committee of the First Affiliated Hospital of Sun Yat-sen University [approval no. (2021):045].

**Animals**

Eight-week-old male Sprague–Dawley rats (250–300 g) were obtained from the Experimental Animal Center of Sun Yat-sen University. Animals were housed under specific pathogen-free conditions in a temperature-controlled environment (22**–**25 °C) with 50**–**60% relative humidity and a 12-h light/dark cycle, with free access to standard chow and water. Rats were randomly assigned to experimental groups prior to surgery. Investigators responsible for outcome assessment were blinded to the group allocation. All animal procedures were approved by the Institutional Animal Care and Use Committee of Sun Yat-sen University (approval no. SYSU-IACUC-2024-001528).

**Intestinal permeability assay**

Following a 6 h fasting period, rats were administered FITC-dextran (5 kDa, 600 mg kg⁻¹) by gavage. Blood samples were collected from the abdominal aorta 4 h later, and plasma was isolated. Fluorescence intensity was measured using a fluorescence microplate reader, and the concentration was calculated based on a standard curve.

**Assessment of gastrointestinal motility**

### Whole gut transit time

Rats were fasted for 6 hours prior to the assay. Phenol red solution (2 mL) was administered orally by gavage, and the time elapsed until the appearance of the first red-colored fecal pellet was recorded. This interval was defined as the whole gut transit time.

### Terminal colonic transit time

Rats were fasted overnight prior to the assay. A smooth plastic bead (approximately 3 mm in diameter) was lightly coated with Vaseline and gently inserted approximately 3 cm into the distal colon using a thin catheter. The interval from insertion to bead expulsion was recorded and defined as the terminal colonic transit time.

### Fecal pellet frequency and water content

On the final day of the experimental intervention, rats were fasted and individually housed in clean cages for 6 hours. All fecal pellets were collected, and the total number of pellets per rat was recorded. Each pellet was weighed to determine the wet weight, then dried at 56 °C overnight to obtain the dry weight. Fecal water content was calculated as the difference between wet and dry weights.

**Real-time quantitative PCR assay**

Total RNA was isolated from collected samples using TRIzol reagent (Molecular Research Center, Inc.) in accordance with the manufacturer’s instructions. RNA concentration and purity were assessed spectrophotometrically using a NanoDrop 8000 system to confirm suitability for subsequent analyses. Equal amounts of RNA (1 μg) were reverse-transcribed into complementary DNA (cDNA) using NovoScript® Plus All-in-One First-Strand cDNA Synthesis SuperMix. Quantitative real-time PCR (qRT-PCR) was carried out using FastStart Essential DNA Green Master Mix (Roche, 06924204001) on a real-time PCR detection platform to determine relative gene expression levels. Primer sequences for all target genes are provided in **Table S1.**

**Western blotting**

Rat tissues or cultured cells were lysed in RIPA buffer supplemented with protease and phosphatase inhibitors after washing with ice-cold PBS. Tissue samples were homogenized prior to lysis. Lysates were clarified by centrifugation at 12,000 rpm for 15 min at 4 °C, and protein concentrations were determined using a BCA assay (Thermo Fisher Scientific). Equal amounts of protein were resolved by SDS–PAGE and transferred onto PVDF membranes. After blocking with 5% bovine serum albumin, membranes were incubated with primary antibodies overnight at 4 °C, followed by incubation with appropriate secondary antibodies for 1 h. Protein signals were quantified using ImageJ software. Antibody details are provided in **Table S2**.

**Immunohistochemistry**

Paraffin-embedded tissue sections were deparaffinized, rehydrated, and subjected to antigen retrieval by high-pressure heating in EDTA buffer. Sections were blocked with 20% normal goat serum for 1 h at room temperature and then incubated with primary antibodies overnight at 4 °C. After washing, the sections were incubated with HRP-conjugated secondary antibodies for 30 min. Immunoreactivity was visualized using DAB and counterstained with hematoxylin. Images were acquired under identical settings. For human rectal specimens, all eligible samples were included, and comparable regions of the muscularis propria were analyzed using ImageJ. Staining was semi-quantified using the H-score method, and the mean score of the analyzed fields was used as the specimen-level value. Representative images were selected from technically adequate sections that reflected the overall staining pattern of the corresponding group, rather than on the basis of unusually strong marker expression. Each marker was evaluated independently on separately stained sections. Antibody details are provided in **Table S2**.

**Immunofluorescence**

Tissue samples were frozen in OCT compound and sectioned at 10–20 μm. Cells cultured on coverslips were fixed under the same conditions. Both tissue sections and cells were fixed with 4% paraformaldehyde for 15 min, permeabilized with 0.5% Triton X-100 for 20 min, and blocked with appropriate serum. Samples were incubated with primary antibodies overnight at 4 °C, followed by fluorophore-conjugated secondary antibodies for 1 h at room temperature. Nuclei were stained with DAPI. Images were captured using a fluorescence or confocal microscope. Antibody details are listed in **Table S2**.

**Rationale for immunohistochemistry and immunofluorescence staining**

Immunohistochemistry and immunofluorescence were selected according to the analytical purpose of each marker. Immunohistochemistry was mainly used for paraffin-embedded sections when chromogenic visualization, tissue-level morphological assessment, and H-score–based semi-quantification were required. Immunofluorescence was used when multi-channel labeling, spatial localization, co-staining, or fluorescence-based readouts were needed, including neuronal fiber networks, synaptic and junctional markers, DHE-based ROS detection, and selected oxidative stress/pyroptosis-related markers. Thus, immunohistochemistry and immunofluorescence were applied as complementary approaches to provide tissue-level validation and spatial fluorescence-based assessment.

**EV isolation and quantification**

For EV collection in the donor-source comparison experiments, Sacral NC, Vagal NC, MSCs, and HDFs were washed twice with sterile PBS and cultured in serum-free collection medium for 36 h. No FBS or other exogenous EV-containing supplements were present during conditioned-medium collection. Conditioned media were collected and centrifuged at 3,000 × g for 15 min to remove cells and debris. EVs were pelleted by ultracentrifugation at 100,000 × g for 70 min at 4 °C, washed once with PBS, and pelleted again under the same conditions. Purified EVs were resuspended in sterile PBS for downstream analyses or functional experiments. Particle concentration and size distribution were measured by nanoparticle tracking analysis after appropriate sample dilution, and the concentrations of the original EV preparations were calculated after correction for the total dilution factor. Relevant experimental information was submitted to the EV-TRACK knowledgebase (EV-TRACK ID: EV260050)[4].

**Transmission electron microscopy**

Freshly isolated EVs were adsorbed onto formvar/carbon-coated copper grids, fixed with 3% glutaraldehyde, and negatively stained with uranyl acetate–methylcellulose. Grids were examined using a transmission electron microscope, and representative images were acquired to assess EV morphology.

**Toluidine blue and Masson trichrome staining**

Paraffin-embedded MPG sections were deparaffinized, rehydrated, and stained with toluidine blue for assessment of general tissue morphology. Adjacent sections were subjected to Masson trichrome staining according to standard protocols to evaluate collagen deposition. Collagen deposition in comparable MPG regions was quantified using ImageJ and expressed as relative collagen content.

**ELISA**

Samples were prepared for enzyme-linked immunosorbent assay (ELISA) by collecting supernatants under defined experimental conditions and clarifying them by centrifugation to remove insoluble material. The levels of IL-18 (Elabscience, Cat. No. E-EL-R0567) and IL-1β (Elabscience, Cat. No. E-HSEL-R0002) were quantified using commercially available ELISA kits according to the manufacturers’ instructions.

**LDH release assay**

PC-12 cells were seeded in 96-well plates and treated as indicated. LDH release was measured using the LDH Cytotoxicity Assay Kit (Beyotime, C0016) according to the manufacturer’s instructions, with absorbance read at 490 nm.

**Proteomic analysis**

Proteomic profiling was performed using three independent biological replicates per condition, comprising three independently prepared Sacral NC-EV samples and three independently prepared HDF-EV samples. EV samples were lysed, and protein concentrations were determined using a BCA protein assay. Equal amounts of protein from each sample were subjected to tryptic digestion, and the resulting peptides were analyzed by nano-liquid chromatography coupled to an Astral high-resolution mass spectrometer in data-independent acquisition (DIA) mode.

Raw mass spectrometry data were processed using DIA-NN, with peptide- and protein-level false discovery rates controlled at <1%. Differential protein abundance between Sacral NC-EVs and HDF-EVs was analyzed using limma. Differentially abundant proteins were subjected to GO enrichment analysis using the EggNOG database, with statistical significance evaluated using Fisher’s exact test.

As a dataset-level assessment independent of candidate prioritization, all identified proteins were compared with ExoCarta and Vesiclepedia human EV protein annotations.

**Candidate prioritization from EV proteomics**

Candidate prioritization was performed using sequential criteria based on differential abundance and consistency across biological replicates. Proteins with nominal P < 0.05 and log₂FC ≥ 1 in Sacral NC-EVs relative to HDF-EVs were retained for initial candidate generation, after which immunoglobulin-related entries were excluded. To minimize the influence of sporadic detection or individual high-abundance preparations, candidates were required to be detected in all three independent Sacral NC-EV preparations and to exhibit a within-group coefficient of variation <0.30. A replicate-wise consistency criterion was then applied, requiring the abundance of each candidate in every Sacral NC-EV preparation to exceed its abundance in every HDF-EV preparation. These criteria defined the reproducibly enriched candidate set used for subsequent phenotype-based prioritization.

The retained candidates were subsequently evaluated for published experimental evidence linking the corresponding protein or its established signaling pathway to NLRP3–caspase-1–GSDMD-mediated pyroptosis. Among these candidates, ANXA1 was prioritized for functional validation because extracellular ANXA1 has been reported to signal through FPR2 and suppress NLRP3 inflammasome-mediated pyroptosis, providing a mechanistic link to the anti-pyroptotic phenotype investigated in this study.

**Transcriptome sequencing and analysis**

Total RNA was extracted from tissue samples. RNA purity was assessed using a NanoDrop 2000 spectrophotometer (OD260/280 ≥ 1.8), and RNA integrity was evaluated using an Agilent 2100 Bioanalyzer. Sequencing libraries were prepared using the Illumina Truseq™ RNA Sample Prep Kit and subjected to paired-end sequencing on the Illumina NovaSeq 6000 platform.

**siRNA-mediated knockdown of *ANXA1***

To generate ANXA1-depleted Sacral NC-EVs, Sacral NC were transfected with small interfering RNA targeting human *ANXA1* or scrambled negative-control siRNA using Lipofectamine 3000 according to the manufacturer’s instructions. Three candidate *ANXA1*-targeting siRNAs were initially screened by qRT-PCR, and the siRNA showing the highest knockdown efficiency was selected for subsequent functional experiments. The sequences of the selected siANXA1 were as follows: sense, 5′-CUCAAGCAGGCCUGGUUUAUUUTT-3′; antisense, 5′-AAUAAACCAGGCCUGCUUGAGTT-3′. The sequences of the scrambled siNC were as follows: sense, 5′-UUCUCCGAACGUGUCACGUTT-3′; antisense, 5′-ACGUGACACGUUCGGAGAATT-3′. At 24 h after transfection, Sacral NC were washed extensively with PBS and cultured in serum-free EV-collection medium for an additional 36 h. Conditioned medium was then collected for EV isolation as described above. EVs derived from siANXA1-transfected Sacral NC were referred to as siANXA1 Sacral NC-EVs, whereas EVs derived from siNC-transfected Sacral NC were referred to as siNC Sacral NC-EVs. Knockdown efficiency was confirmed by qRT-PCR in donor Sacral NC and by western blotting for ANXA1 in the corresponding EV preparations.

**Proteinase K protection assay**

Purified Sacral NC-EVs were normalized by particle number and incubated with Proteinase K (50 μg mL⁻¹) in PBS for 30 min at 37 °C with or without 0.1% Triton X-100. Digestion was terminated with PMSF, followed by western blot analysis using the indicated antibodies. Untreated EVs and Triton X-100–only EVs were included as controls.

**High-salt washing of Sacral NC-EVs**

To assess whether ANXA1 was loosely associated with the EV surface, purified Sacral NC-EVs were subjected to high-salt washing. EV suspensions were incubated in high-salt buffer containing 20 mM HEPES, 1 M NaCl, and 2 mM CaCl₂, pH 7.4, for 30 min at 4 °C with gentle rotation. EVs incubated in PBS under the same conditions were used as controls. After incubation, EVs were recovered by ultracentrifugation at 100,000 × g for 70 min at 4 °C and washed with sterile PBS to remove residual salt. The final EV pellets were lysed in SDS sample buffer and analyzed by western blotting using the indicated antibodies.

**Reconstitution of siANXA1 Sacral NC-EVs with recombinant ANXA1**

siANXA1 Sacral NC-EVs (1 × 10¹⁰ particles) were incubated with 0.1, 0.25, or 0.5 μg recombinant human ANXA1 in annexin-binding buffer (20 mM HEPES, 150 mM NaCl, and 2 mM CaCl₂, pH 7.4) for 30 min at 37 °C[5,6]. EVs were recovered by ultracentrifugation at 100,000 × g for 70 min at 4 °C, washed with PBS, and analyzed by western blotting. The 0.5 μg condition was used for subsequent rescue experiments. Recombinant ANXA1 processed in parallel without EVs served as an EV-free control.

**EV labeling and tracing**

Purified EVs were fluorescently labeled with PKH26 Red Fluorescent Cell Linker dye (Sigma-Aldrich) with minor modifications. Briefly, EVs were incubated with PKH26 in Diluent C at a final dye concentration of 2 μM for 5 min at room temperature in the dark. The labeling reaction was quenched with BSA-containing PBS, and unbound dye was removed by ultracentrifugation and PBS washing. The final PKH26-labeled EV pellets were resuspended in sterile PBS. For dye-control experiments, a PKH26-only control was prepared in parallel using the same labeling, quenching, washing, and ultracentrifugation procedures but without EVs. Unlabeled EVs processed under the same conditions without PKH26 labeling were used as an additional negative control. For in vivo tracing, PKH26-labeled HDF-EVs and Sacral NC-EVs were locally injected into the peri-ganglionic region of the injured MPG using the same source-specific administration volumes and nominal protein inputs as those used in the corresponding therapeutic experiments. Dye-only and unlabeled control preparations were administered at the same injection volume. MPG tissues were harvested 24 h after injection, fixed, cryosectioned, and examined by confocal microscopy under identical imaging settings.

**PC-12 cell culture and H₂O₂ treatment**

PC-12 cells were maintained in RPMI 1640 medium (Gibco) supplemented with 10% fetal bovine serum (FBS; Sigma-Aldrich) and 1% penicillin–streptomycin (Thermo Fisher Scientific) at 37 °C in a humidified atmosphere containing 5% CO₂. Cells were seeded on Matrigel-coated plates and differentiated with 50 ng/mL nerve growth factor (NGF) until neurite outgrowth and neuron-like morphology were observed. For oxidative stress induction, differentiated PC-12 cells were exposed to 100 μM H₂O₂ for 6 h. Cells were treated concurrently with H₂O₂ and either Sacral NC-EVs or HDF-EVs at respective final particle concentrations of approximately 8.6 × 10¹⁰ and 9.7 × 10¹⁰ particles mL⁻¹. For FPR2 blockade experiments, cells were pretreated with BOC-2 or WRW4 (10 μM each) for 30 min before exposure to Sacral NC-EVs and H₂O₂[7,8]. siNC Sacral NC-EVs, siANXA1 Sacral NC-EVs, and ANXA1-reconstituted siANXA1 Sacral NC-EVs were adjusted to particle inputs comparable to that of unmodified Sacral NC-EVs before treatment. For soluble ANXA1 controls, free recombinant human ANXA1 was added directly to H₂O₂-treated cells receiving siANXA1 Sacral NC-EVs at final concentrations of 2, 4, or 6 μg mL⁻¹. Cells were collected after treatment for subsequent analyses.

**References**

1. J. Sun, Y. Huang, J. Gong, et al., “Transplantation of hPSC-Derived Pericyte-Like Cells Promotes Functional Recovery in Ischemic Stroke Mice,” *Nature Communications* 11, no. 1 (2020): 5196, <https://doi.org/10.1038/s41467-020-19042-y>.

2. J. Liu, Z. Li, Q. Ke, et al., “Mesenchymal Stromal Cells Counteract with Age-Related Immune Decline and Enhance Vaccine Efficacy by Modulating Endogenous Splenic Marginal Reticular Cells in Elderly Models,” *Cellular & Molecular Immunology* 23, no. 2 (2026): 220-235, <https://doi.org/10.1038/s41423-025-01381-9>.

3. J. Huang, W. Huang, J. Yi, et al., “Mesenchymal Stromal Cells Alleviate Depressive and Anxiety-Like Behaviors Via a Lung Vagal-to-Brain Axis in Male Mice,” *Nature Communications* 14, no. 1 (2023): 7406, <https://doi.org/10.1038/s41467-023-43150-0>.

4. E.-T. Consortium, J. Van Deun, P. Mestdagh, et al., “EV-TRACK: Transparent Reporting and Centralizing Knowledge in Extracellular Vesicle Research,” *Nature Methods* 14, no. 3 (2017): 228-232, <https://doi.org/10.1038/nmeth.4185>.

5. M. A. Lizarbe, J. I. Barrasa, N. Olmo, F. Gavilanes, and J. Turnay, “Annexin-Phospholipid Interactions. Functional Implications,” *International Journal of Molecular Sciences* 14, no. 2 (2013): 2652-2683, <https://doi.org/10.3390/ijms14022652>.

6. G. I. Perez, M. P. Bernard, D. Vocelle, et al., “Phosphatidylserine-Exposing Annexin A1-Positive Extracellular Vesicles: Potential Cancer Biomarkers,” *Vaccines* 11, no. 3 (2023): <https://doi.org/10.3390/vaccines11030639>.

7. J. Hou, R. Liang, Y. Lin, et al., “Formyl Peptide Receptor 2 (FPR2) Mediates Cisplatin-Induced Cochlear Inflammation and Hair Cell Apoptosis,” *Toxicology* 517 (2025): 154229, <https://doi.org/10.1016/j.tox.2025.154229>.

8. P. Liu, L. Wang, Y. Wang, et al., “ANXA1-FPR2 Axis Mitigates the Susceptibility to Atrial Fibrillation in Obesity Via Rescuing AMPK Activity in Response to Lipid Overload,” *Cardiovascular Diabetology* 23, no. 1 (2024): 452, <https://doi.org/10.1186/s12933-024-02545-z>.

**Supplementary figures**

**Figure S1. Pelvic surgery-associated rectal autonomic denervation and characterization of Sacral NC-EV localization after MPG injury.**

(A,B) Representative immunohistochemical staining of TH (A) and VIP (B) in rectal muscularis propria from Control patients without prior pelvic surgery and Injury patients with a history of pelvic surgery involving pelvic autonomic nerve regions. Scale bars, 100 μm. (C) Semi-quantitative H-score analysis of TH and VIP immunohistochemical staining in the Control and Injury groups; n = 15 independent human specimens per group. (D,E) Representative immunofluorescence staining of TH/TUJ1 (D) and VIP/TUJ1 (E) in rectal tissues from Control and Injury groups. Scale bars, 100 μm. (F) Quantification of relative fluorescence intensity for TH and VIP; n = 15 independent human specimens per group. (G) qPCR analysis of *NANOG*, *SOX10*, and *NGFR* expression in hPSCs and hPSC-derived Sacral NC; n = 3 independent differentiations. (H) qPCR analysis of HOX gene expression, including *HOXB8*, *HOXD8*, *HOXC9*, *HOXB13*, and *HOXD13*, in hPSCs and hPSC-derived Sacral NC; n = 3 independent differentiations. (I) Nanoparticle tracking analysis showing the size distribution of a diluted HDF-EV sample. (J) Representative confocal images showing the relationship between PKH26-labeled Sacral NC-EVs and CD68⁺ macrophages in injured MPG tissues. No obvious colocalization between PKH26-labeled Sacral NC-EVs and CD68⁺ cells was detected. Scale bars, 10 μm. (K,L) Number of fecal pellets excreted within 6 h (K) and fecal water content (L) in Sham, Injury, HDF-EVs, and Sacral NC-EVs groups; n = 5 rats per group. (M) Representative confocal images showing PKH26 signals in MPG tissues after administration of unlabeled EVs, PKH26-only, PKH26-labeled HDF-EVs, or PKH26-labeled Sacral NC-EVs. Unlabeled EVs served as a negative control for PKH26 fluorescence. Scale bars, 20 μm. (N) Quantification of PKH26^+^ area as a percentage of PGP9.5^+^ area in MPG tissues treated with PKH26-labeled HDF-EVs or PKH26-labeled Sacral NC-EVs; n = 3 rats per group. Data are presented as mean ± SD for quantitative panels. In C and F, each dot represents one independent human specimen; in G and H, each dot represents one independent differentiation; in K, L, and N, each dot represents one independent animal. *P* values were calculated using a two-sided Mann–Whitney *U* test for human tissue staining measurements (C,F), a two-sided unpaired Student’s *t*-test for qPCR and EV-distribution comparisons (G,H,N), and one-way ANOVA followed by Tukey’s multiple comparisons test for comparisons among multiple animal groups (K,L). ns, not significant; **P* < 0.05, ***P* < 0.01, ****P* < 0.001.

**Figure S2. Sacral NC-EVs partially improve neuronal subtype innervation in the rectal myenteric plexus following MPG injury.** (A–C) Representative immunofluorescence staining of TUJ1 with nNOS (A), TH (B), or ChAT (C) in rectal myenteric plexus preparations from Sham, Injury, HDF-EVs, and Sacral NC-EVs groups. Scale bars, 100 μm. (D–F) Quantification of nNOS⁺ area fraction (D), TH⁺ area fraction (E), and ChAT⁺ area fraction (F) in the rectal myenteric plexus; n = 3 rats per group. Data are presented as mean ± SD. Each dot represents one independent animal. *P* values were calculated using one-way ANOVA followed by Tukey’s multiple-comparison test. ns, not significant; **P* < 0.05, ***P* < 0.01, ****P* < 0.001.

**Figure S3. Effects of Sacral NC-EVs on TH- and VIP-positive areas in rectal transverse sections after MPG injury.** (A,B) Representative immunofluorescence staining of TUJ1 with TH (A) or VIP (B) in rectal transverse sections from Sham, Injury, HDF-EVs, and Sacral NC-EVs groups. Scale bars, 100 μm. (C,D) Quantification of TH⁺ area as a percentage of the muscularis area (C) and VIP⁺ area as a percentage of the muscularis area (D); n = 5 rats per group. Data are presented as mean ± SD. Each dot represents one independent animal. *P* values were calculated using one-way ANOVA followed by Tukey’s multiple-comparison test. ns, not significant; **P* < 0.05, ***P* < 0.01, ****P* < 0.001.

**Figure S4. Sacral NC-EVs partially preserve mucus barrier integrity and reduce cleaved caspase-3 staining in the rectal mucosa after MPG injury.**

(A) Representative immunofluorescence staining of MUC2 in rectal mucosa from Sham, Injury, HDF-EVs, and Sacral NC-EVs groups. Scale bars, 50 μm. (B) Quantification of relative MUC2 fluorescence intensity; n = 3 rats per group. (C) qPCR analysis of *Muc2* mRNA expression in rectal tissues; n = 3 biological replicates per group. (D) Representative immunofluorescence staining of cleaved caspase-3 in rectal tissues from each group. Following MPG injury, cleaved caspase-3 signal was visually concentrated along the anatomical epithelial region based on tissue morphology and was reduced after Sacral NC-EV treatment. Scale bars, 50 μm. Data are presented as mean ± SD for quantitative panels. Each dot represents one independent animal. *P* values were calculated using one-way ANOVA followed by Tukey’s multiple-comparison test. ns, not significant; **P* < 0.05, ***P* < 0.01.

**Figure S5. Quantification of collagen deposition in MPG tissues after injury and EV treatment.**

(A) Quantification of relative collagen content based on Masson trichrome staining in MPG sections from Sham, Injury, HDF-EVs, and Sacral NC-EVs groups; n = 5 rats per group. Data are presented as mean ± SD. Each dot represents one independent animal. *P* values were calculated using one-way ANOVA followed by Tukey’s multiple-comparison test. ns, not significant; ***P* < 0.01, ****P* < 0.001.

**Figure S6. Sacral NC-EVs attenuate oxidative stress and pyroptosis-associated markers in injured MPG tissues.** (A) Representative immunofluorescence staining of 8-OHdG in MPG tissues from Sham, Injury, HDF-EVs, and Sacral NC-EVs groups. Scale bars, 50 μm. (B) Quantification of 8-OHdG^+^ area fraction; n = 3 rats per group. (C) Representative immunofluorescence staining of NOX2 in MPG tissues from each group. Scale bars, 50 μm. (D) Quantification of NOX2^+^ area fraction; n = 3 rats per group. (E) Representative immunohistochemical staining of GSDMD in MPG tissues from each group. Scale bars, 50 μm. (F–J) qPCR analysis of *Nlrp3* (F), *Casp1* (G), *Gsdmd* (H), *Il18* (I), and *Il1b* (J) mRNA expression in MPG tissues; n = 3 rats per group. (K) Representative immunofluorescence images showing co-staining of GSDMD and PGP9.5 in MPG tissues from the Sham, Injury, and Sacral NC-EV groups. Scale bars, 50 μm. (L) Percentage of PGP9.5-positive neuronal somata exhibiting GSDMD immunoreactivity; n = 3 rats per group. (M) Representative immunofluorescence images showing the spatial relationship of GSDMD immunoreactivity with S100β-positive glial profiles and CD68-positive macrophage profiles in MPG tissues from the Injury group. Scale bars, 50 μm. Data are presented as mean ± SD for quantitative panels. Each dot represents one independent animal. *P* values were calculated using one-way ANOVA followed by Tukey’s multiple-comparison test. ns, not significant; **P* < 0.05, ***P* < 0.01, ****P* < 0.001.

**Figure S7. Sacral NC-EVs attenuate oxidative stress and pyroptosis in H₂O₂-treated PC-12 cells.** (A) Representative DHE staining images showing intracellular ROS levels in PC-12 cells under different treatments. Scale bars, 50 μm. (B) Quantification of relative DHE fluorescence intensity; n = 5 independent experiments. (C) Western blot analysis of pyroptosis-related proteins, including NLRP3, GSDMD-T, GSDMD-N, pro-caspase-1, and cleaved caspase-1, in PC-12 cells under the indicated conditions. (D,E) ELISA analysis of IL-18 (D) and IL-1β (E) levels in cell culture supernatants; n = 3 independent experiments. (F) LDH release assay for evaluation of cell membrane damage; n = 3 independent experiments. Data are presented as mean ± SD for quantitative panels. Each dot represents one independent experiment. *P* values were calculated using one-way ANOVA followed by Tukey’s multiple-comparison test. ns, not significant; ****P* < 0.001.

**Figure S8. Sacral NC-EVs exhibit distinct antioxidative, anti-pyroptotic, and neuronal effects compared with MSC-EVs and Vagal NC-EVs.** (A) Representative immunofluorescence staining of HOXB4, SOX10, and NGFR in hPSC-derived Vagal NC. Scale bars, 50 μm. (B) qPCR analysis of *NANOG*, *SOX10*, and *NGFR* expression in hPSCs and Vagal NC; n = 3 independent differentiations. (C) qPCR analysis of HOX gene expression in hPSCs and Vagal NC, showing *HOXB3* and *HOXB5* expression in Vagal NC; n = 3 independent differentiations. (D) Western blot analysis of EV-positive markers CD9, CD81, and TSG101 and the negative marker CALNEXIN in MSCs, MSC-EVs, Vagal NC, and Vagal NC-EVs. (E) Western blot analysis of source-associated markers in HDF-EVs, MSC-EVs, Vagal NC-EVs, and Sacral NC-EVs, including CD73, Vimentin, FN1, NGFR, and CD81. (F–K) Quantitative analysis of immunofluorescence staining in MPG tissues from Injury, MSC-EVs, Vagal NC-EVs, and Sacral NC-EVs groups, including relative DHE fluorescence intensity (F), GSDMD^+^ area fraction (G), PGP9.5^+^ area fraction (H), PSD-95^+^ area fraction (I), ChAT^+^ area fraction (J), and nNOS^+^ neuron density (K); n = 3 rats per group. Data are presented as mean ± SD for quantitative panels. In B and C, each dot represents one independent differentiation; in F–K, each dot represents one independent animal. *P* values were calculated using a two-sided unpaired Student’s *t*-test for two-group qPCR comparisons (B,C) and one-way ANOVA followed by Tukey’s multiple comparisons test for comparisons among multiple animal groups (F–K). ns, not significant; **P* < 0.05, ***P* < 0.01, ****P* < 0.001.

**Figure S9. EV-associated ANXA1 supports the antioxidant and anti-pyroptotic effects of Sacral NC-EVs through FPR2 signaling.** (A) Western blot analysis of ANXA1 expression in Sacral NC-EVs, HDF-EVs, MSC-EVs, and Vagal NC-EVs. (B) Western blot analysis of ANXA1 in Sacral NC-EVs before and after high-salt washing to assess the association of ANXA1 with EVs after removal of loosely attached surface proteins. (C) qPCR analysis of *ANXA1* expression in control, siNC-treated, and siANXA1-treated Sacral NC; n = 3 independent experiments. (D) Dose screening of recombinant ANXA1 for reconstitution of siANXA1 Sacral NC-EVs. siNC Sacral NC-EVs and siANXA1 Sacral NC-EVs were used as reference controls. Recombinant ANXA1 was incubated with siANXA1 Sacral NC-EVs at the indicated amounts, followed by ultracentrifugation and washing to remove unbound protein. (E,F) Representative DHE fluorescence images (E) and quantification of relative DHE fluorescence intensity (F) in H₂O₂-treated PC-12 cells treated with siANXA1 Sacral NC-EVs, Sacral NC-EVs combined with the FPR2 antagonist WRW4, ANXA1-reconstituted siANXA1 Sacral NC-EVs (EV-rANXA1), or free recombinant ANXA1 at 2, 4, or 6 μg/mL. n = 4 independent experiments. Scale bars, 100 μm. (G,H) Quantification of relative DHE fluorescence intensity (G) and GSDMD^+^ area fraction (H) in MPG tissues from different treatment groups; n = 3 rats per group. (I,J) Representative immunofluorescence staining (I) and quantification (J) of 8-OHdG^+^ area fraction in MPG tissues from the indicated groups; n = 3 rats per group. (K,L) Representative immunofluorescence staining (K) and quantification (L) of NOX2^+^ area fraction in MPG tissues from the indicated groups; n = 3 rats per group. Scale bars, 50 μm. Data are presented as mean ± SD for quantitative panels. In C and F, each dot represents one independent experiment; in G, H, J, and L, each dot represents one independent animal. *P* values were calculated using one-way ANOVA followed by Tukey’s multiple-comparison test. ns, not significant; **P* < 0.05, ***P* < 0.01, ****P* < 0.001.

**Supplementary tables**

**Table S1: List of primers used for qRT-PCR.**

| **Gene** | **Sequences (5′ to 3′)** | **Application** |
| --- | --- | --- |
| *Tjp1* | Forward: GGGGAAACCCGAAACTGATGC | qPCR |
|  | Reverse: GTGGAGAGAAGAGTTGGACAGAGGC |  |
| *Ocln* | Forward: GGGACAGAGCCTATGGAACG | qPCR |
|  | Reverse: CCAAGGAAGCGATGAAGCAA |  |
| *Cldn1* | Forward: ATTTCAGGTCTGGCGACATTAGTGG | qPCR |
|  | Reverse: ACAGGAGCAGGAAAGTAGGGCAC |  |
| *Muc2* | Forward: GATAGGTGGCAGACAGGAGA | qPCR |
|  | Reverse: GCTGACGAGTGGTTGGTGATTG |  |
| *Nlrp3* | Forward: TGGGGGACTTTGGAGTCAGA | qPCR |
|  | Reverse: GCAGCTGACCAACCAGAGTT |  |
| *Casp1* | Forward: GAAACGCCATGGCTGACAAG | qPCR |
|  | Reverse: TCCTGGTTCAGGACTCTCTTCT |  |
| *Il1b* | Forward: CAGCTTTCGACAGTGAGGAGA | qPCR |
|  | Reverse: TGTCGAGATGCTGCTGTGAG |  |
| *Il18* | Forward: CAGCTCTTCTACCAGCAAACAT | qPCR |
|  | Reverse: CTTCCAACTGAGAGGCTGTGC |  |
| *ANXA1* | Forward: CAGGTCACCTTGAGGAGGTTGT | qPCR |
|  | Reverse: CAGCACGAAGTTCATCAGCATC |  |
| *Gsdmd* | Forward: GGGATTCAGCACTTCCCAGAA | qPCR |
|  | Reverse: GCTGACGGCATGATCCTCTTAG |  |
| *Gapdh* | Forward: CAAGGTCATCCATGACAACTTTG | qPCR |
|  | Reverse: GGCCATCCACAGTCTTCTGG |  |
| *SOX10* | Forward: CCTCACAGATCGCCTACACC | qPCR |
|  | Reverse: CATATAGGAGAAGGCCGAGTAGA |  |
| *NGFR* | Forward: CGGGCCAACATGCAAAAGTC | qPCR |
|  | Reverse: CAGATGCGGTTCTGTTCCC |  |
| *NANOG* | Forward: CAGCCCAGATTCTTCCACCAGTCCC | qPCR |
|  | Reverse: CGGAAGCGTTCCCAGTCGGGTTCACC |  |
| *HOXB13* | Forward: AGCTCCCGTGCCTTATGGTTA | qPCR |
|  | Reverse: GGCTGGTAGGTTCCCGGATA |  |
| *HOXD13* | Forward: CTTCGGCAACGGCTACTACAG | qPCR |
|  | Reverse: TGACACGTCCATGTACTTCTCC |  |
| *HOXB3* | Forward: CCAGTGCCACTAGCAACAG | qPCR |
|  | Reverse: CGTTTGCCTCGACTCTTTCATC |  |
| *HOXB5* | Forward: AACTCCTTCTCGGGGCGTTAT | qPCR |
|  | Reverse: CATCCCATTGTAATTGTAGCCGT |  |
| *HOXB8* | Forward: GTCCCTGCGCCCCAATTATTA | qPCR |
|  | Reverse: GCCCGTGGTAGAACTCCTG |  |
| *HOXD8* | Forward: AGAAGAATCGAGGTTTCCCACG | qPCR |
|  | Reverse: TCCTTTTTCGTTTCCCCGTCC |  |
| *HOXC9* | Forward: AGCACAAAGAGGAGAAGGC | qPCR |
|  | Reverse: CGTCTGGTACTTGGTGTAGG |  |
| *GAPDH* | Forward: ATTCCATCCCAGACCCCATAAC | qPCR |
|  | Reverse: GCAGCGAACTTTATTGATGGTAT |  |

**Table S2: Detailed information of the antibodies used in this study.**

| Antigen | Manufacturer | Catalog Number | Application |
| --- | --- | --- | --- |
| TUJ1 | R&D Systems | MAB1195 | 1:1000 for IF  1:1000 for IHC |
| Caspase-3 | Proteintech | 19677-1-AP | 1:200 for IF  1:1000 for WB |
| ChAT | Proteintech | 24418-1-AP | 1:100 for IF  1:200 for IHC |
| TH | Millipore | AB152 | 1:100 for IF  1:500 for IHC |
| VIP | Novus | NBP1-05163 | 1:100 for IF  1:100 for IHC |
| nNOS | Abcam | Ab76067 | 1:200 for IF  1:200 for IHC |
| ANXA1 | Abcam | Ab214486 | 1:500 for WB |
| S100β | Proteintech | 15146-1-AP | 1:100 for IF |
| NeuN | Proteintech | CL488-26975 | 1:200 for IF |
| ZO-1 | Proteintech | 66452-1-Ig | 1:1000 for WB  1:200 for IF |
| Occludin | Proteintech | 27260-1-AP | 1:5000 for WB  1:200 for IF |
| CD31 | Abcam | Ab222783 | 1:100 for IHC |
| nNOS | SantaCruz | sc-5302 | 1:100 for IF |
| SOX10 | Abcam | Ab155279 | 1:500 for IF |
| HOXD13 | Proteintech | 23520-1-AP | 1:100 for IF |
| Fibronectin (FN1) | Proteintech | 66042-1-Ig | 1:2000 for WB |
| Claudin-1 | Proteintech | 13050-1-AP | 1:1000 for WB  1:1000 for IF |
| MUC2 | Proteintech | 27675-1-AP | 1:200 for IF |
| BAX | Proteintech | 50599-2-Ig | 1:10000 for WB |
| Bcl-2 | Cell Signaling Technology | 2876 | 1:1000 for WB |
| VEGF | Abcam | ab52917 | 1:300 for IF |
| GSDMD | SantaCruz | sc-393581 | 1:100 for IF |
| Cleaved caspase-3 | Abcam | ab32042 | 1:100 for IF |
| PSD-95 | Cell Signaling Technology | 3450T | 1:100 for IF |
| Phospho-CREB | Cell Signaling Technology | 9198T | 1:400 for IF |
| Synaptophysin | Abcam | ab32127 | 1:100 for IF |
| 8-OHdG | SantaCruz | sc-66036 | 1:100 for IF |
| PGP9.5 | Proteintech | 66230-1-Ig | 1:200 for IF |
| Pro Caspase-1 | Abcam | ab179515 | 1:1000 for WB |
| NOX2 | Proteintech | 19013-1-AP | 1:200 for IF |
| Cleaved GSDMD | Cell Signaling Technology | 10137 | 1:1000 for WB |
| Cleaved Caspase-1 | Cell Signaling Technology | 89332 | 1:1000 for WB |
| GSDMD | Proteintech | 20770-1-AP | 1:200 for IF  1:200 for IHC |
| NLRP3 | Zenbio | R381207 | 1:100 for IHC  1:500 for WB |
| HOXB4 | Abcam | ab133521 | 1:100 for IF |
| NGFR | Abcam | ab52987 | 1:200 for IF |
| CD68 | Abcam | ab125212 | 1:200 for IF |
| CD9 | Abcam | [ab236630](https://www.abcam.cn/products/primary-antibodies/cd9-antibody-epr23105-121-ab236630) | 1:1000 for WB |
| CD81 | Proteintech | 27855-1-AP | 1:1000 for WB |
| TSG101 | Proteintech | 28283-1-AP | 1:1000 for WB |
| CALNEXIN | Abcam | [ab22595](https://www.abcam.cn/products/primary-antibodies/calnexin-antibody-er-marker-ab22595) | 1:1000 for WB |
| ALIX | Abcam | [ab275377](https://www.abcam.cn/products/primary-antibodies/alix-antibody-epr23653-32-ab275377) | 1:1000 for WB |
| CD73 | Abcam | [ab133582](https://www.abcam.cn/products/primary-antibodies/cd73-antibody-epr6114-ab133582) | 1:1000 for WB |
| Vimentin | Proteintech | 10366-1-AP | 1:20000 for WB |
| β-actin | Proteintech | 66009-1-Ig | 1:10000 for WB |
| anti-mouse IgG HRP-linked Ab | Cell Signaling Technology | 7076 | 1:1000 for WB |
| anti-rabbit IgG HRP-linked Ab | Cell Signaling Technology | 7074 | 1:1000 for WB |
| goat anti-mouse IgG Alexa 488 | Invitrogen | A11001 | 1:500 for IF |
| goat anti-rabbit IgG Alexa 488 | Invitrogen | A11008 | 1:500 for IF |
| goat anti-rabbit IgG Alexa 555 | Invitrogen | A21428 | 1:500 for IF |
| goat anti-mouse IgG Alexa 555 | Invitrogen | A21422 | 1:500 for IF |
